# Characterization of cold plasma-induced covalent epigallocatechin gallate conjugates of β-lactoglobulin: a comparison with free-radical and alkaline treatments

**DOI:** 10.1016/j.fochx.2025.103330

**Published:** 2025-12-01

**Authors:** Zhi-Wei Liu, Jun Lv, Chang Liu, Jun-Hu Cheng, Najla AlMasoud, Rana Muhammad Aadil, Xiu-Bin Liu

**Affiliations:** a College of Food Science and Technology, Hunan Agricultural University, Changsha 410128, China; b School of Food Science and Engineering, South China University of Technology, Guangzhou 510641, China; c Guangdong Key Laboratory of Food Intelligent Manufacturing, Foshan University, Foshan 528225, China; d Changsha Innovation Institute for Food, Changsha 410128, China; e Department of Chemistry, College of Science, Princess Nourah bint Abdulrahman University, PO Box 84428, Riyadh 11671, Saudi Arabia; f National Institute of Food Science and Technology, University of Agriculture, Faisalabad 38000, Pakistan

**Keywords:** β-lactoglobulin, Epigallocatechin gallate, Cold plasma, Free radical, Alkaline treatment, Covalent conjugates, Functional food

## Abstract

The characteristics of cold plasma (CP)-induced β-lactoglobulin-epigallocatechin gallate (β-LG-EGCG) covalent conjugates were investigated in comparison with the conventional free -radical (FR) and alkaline treatment (AT) methods. Results indicated CP achieved a high EGCG grafting degree (116.73 ± 0.82 μmol/g, equivalent to 38.91 ± 0.27 % of grafting) after 45 s treatment, comparable to AT (131.79 ± 1.75 μmol/g, equivalent to 43.93 ± 0.58 % of grafting) and far exceeding FR (18.29 ± 0.67 μmol/g, equivalent to 6.10 ± 0.22 % of grafting). In addition, CP conjugates exhibited 27.51 % less browning than AT counterparts. Different from AT and FR, CP uniquely expanded EGCG-binding sites to include hydroxyl- and cyclic alkyl-containing residues. Multiple structural analyses revealed covalent grafting with EGCG induced conformational unfolding of β-LG and exposed buried hydrophobic groups. The CP-induced β-LG-EGCG conjugates yielded improvements comparable to AT in emulsifying and antioxidant properties. Moreover, CP-induced conjugates exhibited superior digestibility and lower antigenicity during gastrointestinal digestion. These findings demonstrated CP is a robust means of promoting covalent grafting between proteins and polyphenols, with high efficiency and enhanced functionality.

## Highlights

• Comparable epigallocatechin gallate grafting degree obtained after 45 s of CP treatment, compared to AT.

• 27.51 % reduction in browning for CP-induced conjugates relative to AT counterparts.
• CP expanded epigallocatechin gallate-binding sites to hydroxyl- and cyclic alkyl-containing residues.
• CP-induced conjugates yielded improvements comparable to AT in emulsifying and antioxidant properties.
• CP-induced conjugates exhibited superior digestibility and lower antigenicity.

## 1 Introduction

Interactions between proteins and polyphenols represent a ubiquitous phenomenon in foods, particularly evident in tea-milk systems and berry-containing dairy products (Yan et al., 2023). This interaction has attracted considerable attention due to improvements in protein functional properties (e.g., emulsifying, foaming, antioxidant and gelation properties) as well as increased digestibility, reduced antigenicity, and enhanced stability, bioaccessibility and bioavailability of polyphenols (Kradetskaya et al., 2024; Wang et al., 2025). The interaction between proteins and polyphenols can be classified into non-covalent and covalent interactions. Recently, research focus has shifted toward covalent conjugation strategies due to their enhanced stability under food-processing conditions (e.g., heating and pH adjustment) (Liu et al., 2024). Conventionally, covalent protein-polyphenol conjugates can be formed using enzymatic methods (e. g., tyrosinase and laccase), free-radical methods (e. g., H_2_O_2_ + ascorbic acid), and alkaline treatment method (pH 9) (Yan et al., 2023). Enzymatic methods have become less common due to operational complexity and high cost (Feng et al., 2023). The covalent linkages predominantly involve Michael-addition adducts (C—N), Schiff-base formations (C=N), and thiol-quinone couplings (C—S). Electrophilic o-quinone structures (generated in alkaline and enzymatic methods) formed by polyphenol oxidation, and oxidation of protein amino-acids side-chain groups (e.g., -SH, -NH_2_ and -NH; in free-radical methods), are involved in covalent crosslinking between proteins and polyphenols (Yan et al., 2023). Unlike non-covalent interactions (hydrogen bonding, ionic, hydrophobic, and van der Waals forces) with reversible binding, covalent protein-polyphenol conjugates exhibit high structural robustness, maintaining integrity under harsh processing conditions (Feng et al., 2023). Nevertheless, conventional covalent conjugation methods that require extended reaction periods (>24 h) present critical challenges: (1) elevated risk of microbial contamination and (2) noncompliance with contemporary clean-label standards, collectively limiting their industrial implementation.

Recently, emerging physically assisted techniques, including ultrasound, pulsed electric fields, high hydrostatic pressure, and magnetic fields, have demonstrated efficacy in accelerating protein-polyphenol covalent conjugation and reducing conventional processing times (Chen, Wei, Chen, Feng, & Zhang, 2024; Han et al., 2024; Rueangsri, Lasunon, Kwantrairat, & Taweejun, 2025). However, these approaches remain fundamentally constrained by their reliance on traditional reaction mechanisms, necessitating further innovation to address persistent limitations in scalability and process efficiency. Cold plasma (CP), an innovative non-thermal processing technology, has emerged as a sustainable alternative to conventional protein-polyphenol conjugation methods (Liu et al., 2025b). Our prior studies demonstrate CP's unique advantages: (1) rapid covalent conjugation within 60 s without chemical reagents, (2) dose-dependent improvements in antioxidant, and emulsifying properties, and (3) reduced antigenicity of food-protein allergens (e.g., 63.13 % IgE reduction in ovalbumin-polyphenol conjugates) (Liu, Tang, et al., 2025b). Specifically, CP-induced β-LG-EGCG conjugates achieved 102 μmol/g EGCG incorporation (45 s), with 8-fold higher DPPH and 2-fold greater ABTS scavenging activity compared with native β-LG, alongside a 29.97 % IgG reduction (Lv et al., 2024). Despite its established the efficacy of CP in promoting polyphenol grafting and enhancing protein functionality, crucial gaps remain regarding the properties of CP-induced conjugates, such as digestibility, and post-digestion antigenicity. Moreover, a comprehensive comparative analysis against established alkaline and free-radical methods, especially in terms of binding degree, site specificity, and these functional attributes, has not been conducted.

Therefore, this study aims to elucidate the differences in the characteristics of CP-induced β-LG-EGCG covalent conjugates relative to conventional FR and AT methods. The formation of β-LG-EGCG covalent conjugates was confirmed by SDS-PAGE, LC-MS/MS, and quantification of EGCG content. The EGCG binding sites in β-LG-EGCG conjugates were identified by LC-MS/MS. UV–vis, circular dichroism (CD), intrinsic fluorescence, and surface hydrophobicity analyses were used to assess structural changes of β-LG after conjugation with EGCG. Browning degree and water contact angle measurements were used to evaluate the browning and wettability of the conjugates. In addition, the functional properties of the conjugates (DPPH and ABTS radical scavenging abilities, and emulsifying properties) were evaluated. Finally, the digestibility and the IgG/IgE binding abilities of the conjugates before and after digestion were measured.

## 2 Materials and methods

### 2.1 Materials and chemicals

β-LG (purity ≥95 %) was purchased from Aladdin Biotech Co., Ltd. (Shanghai, China). EGCG (purity ≥99 %) were provided by Macklin Co., Ltd. (Shanghai, China). The supplier of pepsin (≥ 2500 U/mg) and Trypsin (≥ 1500 U/mg) was Sigma (St. Louis, MO, USA). In our laboratory, rabbits were immunized rabbits with β-LG to generate a β-LG antibody. HRP-labeled goat anti-rabbit IgG labeled was supplied by Sangon Biotech (Shanghai, China). Sera from 14 milk-allergic patients (Table S1) were collected at Yi Yang Center Hospital, with approval from the Medical Ethics Committee of Hunan Agricultural University (File No. 2021036). HRP-labeled goat anti-human IgE was obtained from Thermo Fisher (MA, USA). All other chemicals used were of reagent grade.

### 2.2 β-LG-EGCG conjugates preparation

Free-radical, alkaline and cold plasma treatment was used to prepare β-LG-EGCG covalent conjugates according to Wu et al. (2018), Man et al. (2024) and Lv et al. (2024), with slight modifications.

Free-radical method: A 2 mg/mL β-lactoglobulin (β-LG) solution was prepared in deionized water, followed by sequential addition of H_2_O_2_ (0.1 M, final concentration) and ascorbic acid (5 mg/mL, final concentration). After 2 h of incubation at room temperature (25 °C), EGCG (0.6 mM, final concentration) was introduced, and the reaction proceeded for 24 h at 25 °C with constant agitation (200 rpm).

Alkaline and cold plasma treatment method: β-lactoglobulin (β-LG; 2 mg/mL) and EGCG (0.6 mM) were co-dissolved in deionized water to prepare the reaction mixture. For cold plasma treatment, Air was used as feeding gas. Samples were exposed to dielectric-barrier-discharge atmospheric cold plasma (DBD-ACP) using a CTP-2000 K plasma generator (Nanjing Suman Electronics Co., Ltd., China) under optimized parameters (40 kV, 13 kHz, 45 s), followed by immediate storage at 4 °C. For alkaline conjugation, the mixture pH was adjusted to 9.0 (1 M NaOH) and incubated at 25 °C for 24 h with continuous agitation (200 rpm).

To remove non-covalently bound EGCG, all samples were dialyzed (MWCO: 8–14 kDa, 4 °C, 48 h), lyophilized (SCIENTZ-18 freeze-dryer, Ningbo Scientz Biotechnology), and stored at 4 °C. Control samples were prepared identically without free-radical induction, alkaline treatment, cold plasma exposure, or EGCG addition. The resulting covalent β-LG-EGCG conjugates were designated as FR (free radical grafted), AT (alkaline treated), and CP (cold plasma processed).

### 2.3 SDS-PAGE analysis

SDS-PAGE analysis was performed following our previous studies (Lv et al., 2024) with slight modification. Protein samples (1 mg/mL in 0.1 M PBS, pH 7.0) were mixed with reducing (4:1 *v/v*) or non-reducing (3:1 *v/v*) loading buffers, denatured at 90 °C for 10 min, and resolved using a discontinuous system (5 % stacking/15 % separating gels). Aliquots (8 μg protein/lane) and protein markers (5 μL/lane) were electrophoresed at 75 V for 3 h, followed by Coomassie staining and destaining.

### 2.4 EGCG content analysis

The concentration of EGCG in β-LG-EGCG conjugates was determined by a modified Folin-Ciocalteu colorimetric method (Chen et al., 2023). 0.5 mL aliquots of the sample solution (1 mg/mL prepared in 0.1 M phosphate buffer, pH 7.0) were placed in centrifuge tubes, then 2.5 mL of 0.2 M Folin-Ciocalteu reagent was added, the sample was vortexed, and incubated in the dark at 25 °C for 5 min. To the tubes containing the Folin-Ciocalteu reagent, 2 mL of sodium carbonate (75 mg/mL) was added, and the reaction mixture was then placed in the dark for 2 h. Afterwards the absorbance was recorded at 760 nm using a UV1901G spectrophotometer (Shanghai Yoke Instrument). The EGCG content and degree of EGCG grafting was calculated with the following Eq. (1) and Eq. (2), respectively.

(1) $$\text{EGCG content}\;\left(\text{μmol}/g\right)=\frac{C_{1}\times 10^{6}}{\left(1-C_{1}\right)\times 458}$$

(2) $$\text{Degree of EGCG grafting}\;\left(\%\right)=\frac{C_{1}\times C_{2}}{\left(1-C_{1}\right)\times C_{3}}\times 100\%$$

where C_1_ mean the concentration of EGCG (mg/mL) in β-LG-EGCG conjugates (1 mg/mL) calculated by standard calibration curve (Table S2). C_2_ (2 mg/mL) and C_3_ (0.2748 mg/mL, equivalent to 0.6 mM) is the concentration of β-LG and EGCG in β-LG-EGCG conjugates preparation (Section 2.2), respectively. The molecular weight of EGCG is 458 g/mol and 10^6^ is conversion factor.

### 2.5 Determination of browning degree

The browning degree of sample solutions (1 mg/mL in 0.1 M PBS, pH 7.0) was assessed by measuring absorbance at 432 nm, which represented the maximum absorption wavelength of samples in range of 400–1100 nm.

### 2.6 LC-MS/MS analysis

The LC-MS/MS analyses were conducted on a Q Exactive™ HF-X Hybrid Quadrupole-Orbitrap Mass Spectrometer interfaced with an Ultimate 3000 UHPLC system (Thermo Fisher Scientific, USA). The β-LG-EGCG conjugates were biochemically digested: first with pepsin (for hydrochloric acid denaturing) and then enzymatically with trypsin and chymotrypsin according to protocols by Wiśniewski, Zougman, Nagaraj, and Mann (2009). The peptide mixtures were fractionated on an EASY-nLC 1200 (Thermo Fisher Scientific, USA) with 2 solvents: Mobile phase A (formed by 0.1 % *v*/v formic acid in water) and Mobile phase B (0.1 % v/v formic acid in 84 % v/v acetonitrile). The peptide ligations were first trapped and separated in a Thermo Scientific EASY-Spray trapping column (100 μm × 2 cm, 5 μm C18) pre-equilibrated with 95 % Mobile phase A, and then transferred to an analytical column (75 μm × 10 cm, 3 μm C18). The system operated at a flow of 250 nL/min. A gradient program was then set up in sequence: 0–50 min at 4–50 % Mobile phase B, 50–54 min to 100 % Mobile phase B, and 54–60 min sustained at 100 % Mobile phase B. The melted ligated peptides were ionized in positive form and then analyzed over a 60 min sequence on Q Exactive™ HF-X. A set of full-scan MS and MS/MS fragment spectra was obtained in which a peptide and it's fragments were analyzed to determine mass-to-charge value. Data processing was carried out in MaxQuant (version 2.1.1.1) using targeted searches against the β-LG protein database. The non-fixed mode was used, and the cutoff for the FDR value is 0.01.

### 2.7 Structure changes analysis

#### 2.7.1 Circular dichroism UV spectroscopy

The secondary structural changes of β-LG attached to EGCG covalently were studied using a J-1500-150 circular dichroism spectrometer (Jasco, Tokyo, Japan). The conjugate solutions were made with 10 mM PBS (pH 7.0) with a final concentration of 0.012 mg/mL. The range of 190–260 nm was scanned for spectra, with a data pitch of 1.0 nm, a scanning speed of 100 nm/min, and a bandwidth of 1.00 nm using a quartz cuvette with a 1 cm path length. The measurements were done in triplicate for each sample. The resulting spectra were analyzed with CDNN software to estimate the proportions of α-helix, β-sheet, β-turn, and random coil structures (Lei et al., 2023).

#### 2.7.2 UV–vis absorption spectra

The UV–vis absorption spectrum of samples (1 mg/mL, 0.1 M PBS, pH 7.0) was measured by a UV1901G spectrometer over 190–400 nm.

#### 2.7.3 Intrinsic fluorescence analysis

Samples (1 mg/mL), and their intrinsic tryptophan fluorescence, were examined on an F-7000 fluorescence spectrophotometer (Hitachi High-Tech, Kyoto, Japan). The spectrophotometer settings were configured for the excitation light to be 280 nm, while the emission spectra were collected and scanned between 300 and 400 nm at a rate of 2400 nm/min during the spectrophotometer emission scanning. Slits for both excitation and emission were set to 2.5 nm.

#### 2.7.4 Surface hydrophobicity analysis

The quantification of β-LG surface hydrophobicity changes after conjugation of β-LG with EGCG was performed using the fluorescent probe 8-anilino-1-naphthalenesulfonate (ANS). The stock solution of ANS (5 mM) was made in 0.1 M PBS and 10 μL of this solution was diluted with 1 mL of the samples (1 mg/mL). The sample and ANS solutions were kept in the dark at room temperature for 30 min with intermittent shaking before being analyzed. The fluorescence spectrum was recorded with excitation of 380 nm and emission slit width of 5 nm with the emission scans being captured between 400 and 600 nm. The scans were executed at a speed of 2400 nm/min and the slit width of the excitation beam was 2.5 nm.

### 2.8 Water contact angle (WCA)

As written and reported, the sample water contact angle measurement aimed to determine the sample wettability by surface tension (Z. Li, Deng, & Chen, 2022). The droplets of distilled water, with the volume of 5.0 μL, were deposited with the help of a micro-syringe onto the surface of the sample that had been pressed into sheets of the certain dimension. The optical contact angle measuring device (LSA 100, Lauda Scientific, Germany) was used to determine the contact angle.

### 2.9 Functionalities analysis

#### 2.9.1 Antioxidant capacity

The ABTS and DPPH radical scavenging activities of β-LG and β-LG-EGCG conjugates were conducted as per Liu, Tang, et al. (2025b). And for the analysis, the Trolox calibration curve was used (Table S3).

#### 2.9.2 Emulsifying properties

The β-LG and its conjugates were tested for emulsifying activity and emulsifying stability indexes (EAI and ESI, respectively) using the methods described by (Pan et al., 2023). Six milliliters of sample solution (1 mg/mL in 0.1 M PBS, pH 7.0) was added to a glass vial of 2 mL soy oil for each measurement. The emulsion was obtained by treating the sample in a high-speed shear homogenizer (THF500-12G, Tuohe Electromechanical Technology Co. Ltd., Shanghai, China) for 2 min at 12,000 rpm. Immediately after preparation, 50 μL of the emulsion was diluted to 4 mL with 0.1 % sodium dodecyl sulfate (SDS) solution, and the absorbance at 500 nm was measured using a UV–Vis spectrophotometer.

(3) $$\mathrm{EAI}\;\left(m^{2}/g\right)=\frac{2\times 2.303\times A_{0}\times D}{C\times \left(1-V\right)\times 10000}$$

(4) $$\mathrm{ESI}\;\left(\min\right)=\frac{A_{10}}{A_{0}}\times 100$$

where A_0_ and A_10_ refer to the absorbance values of emulsion at time 0 and time 10 min respectively, D is the dilution factor set to 80 (80 = 4000/50), C is the protein concentration set to 0.001 g/mL, and V is the oil volume fraction set at 0.25.

### 2.10 In vitro digestibility analysis

#### 2.10.1 In vitro simulated gastrointestinal digestion

The study of in vitro gastrointestinal digestion of conjugates of β-LG and β-LG-EGCG was done following the works of Hao et al. (2022). Briefly, the simulated gastric fluid (SGF) and simulated intestinal fluid (SIF) were prepared as described by Hao et al. (2022). For gastric digestion, 10 mL of sample solution (1 mg/mL, distilled water) was mixed the SGF and SIF were then prepared. For the gastric digestion, the sample was blended with the SGF in the ratio 1:1 and then the pepsin, with the enzyme activity of 2000 U/mL, was added to the solution. For neutralizing the reaction, the pH was adjusted to 7.0 with 1 M NaOH. For the intestinal phase, the gastric digesta was combined with SIF at the ratio of 1:1 with the addition of trypsin, bile salts, and CaCl_2_. The digestion was then stored at 4 °C and analyzed later on***.***

#### 2.10.2 Tricine SDS-PAGE

Tricine SDS-PAGE analysis of gastric and gastrointestinal digesta was adapted from Wang et al. (2021). Tricine SDS-PAGE gels were prepared using a commercially available kit (Tricine SDS-PAGE gel preparation kit, Beyotime Biotechnology Co., Ltd., Shanghai, China). Samples were diluted 1:1 (*v/v*) with loading buffer, and incubated at 90 °C for 10 min. Electrophoresis was performed on gels comprising a 4 % stacking gel and a 16 % separating gel. A total of 20 μL of each sample and 5 μL of protein marker were loaded. The electrophoresis at 30 V (1 h) followed by 120 V (3 h) in Tris-Tricine-SDS buffer, with subsequent Coomassie staining and destaining.

#### 2.10.3 Protein digestibility

Protein digestibility was analyzed by the trichloroacetic acid (TCA) precipitation method according to Zhang et al. (2021), with some modifications. To precipitate proteins, 1.5 mL of each digested and non-digested sample was mixed with an equal volume (1:1, *v/v*) of 10 % TCA solution and thoroughly vortexed. The resulting mixtures were refrigerated overnight at 4 °C and then centrifuged at 10000 rpm for 20 min. After discarding the supernatants, the protein precipitates were reconstituted in 1 mL of 1 M NaOH. Protein concentration was subsequently determined using a Bradford assay kit. Protein digestibility was then calculated using the following Eq. (3):

(5) $$\text{Digestibility}\;\left(\%\right)=\frac{C_{0}-C_{T}}{C_{0}\;}\times 100$$

where C_0_ and C_T_ represent the concentrations of the total and TCA-precipitated proteins, respectively. The group containing only water served as the blank.

### 2.11 Antigenicity of β-LG-EGCG conjugates and of its digested samples

The binding capacity of β-LG-EGCG conjugates and their digested products toward IgE and IgG was evaluated using an ELISA assay (Wang et al., 2021). To each 96 well polystyrene plate, 100 μL of the diluted sample (to 25 μg/mL in carbonate buffer, 50 mM, pH 9.6) was added. To coat the antigen, the plates were kept at 4 °C overnight and after that, the plates were washed 3 times with TBST.

Each well received a coating of blocking solution, in this case, 250 μL, for 1 h at 37 °C. The blocking solution was then removed, and the wells were washed. Patients' sera obtained from the milk-sensitized women, 1:2 diluted, and the rabbit anti-β-LG antibody (IgG, 1:1000) were added as the primary antibodies at a 100 μL volume per well. The wells were then washed, and the secondary antibodies were added as follows: at a 100 μL volume per well, the goat anti-human IgE HRP conjugate (1:1000 dilution) and HRP goat anti-rabbit IgG (1:20,000 dilution) were added. Following careful washing, 100 μL of TMB substrate from the Beyotime Biotechnology Co. was added to each well. The solution was then incubated at 37 °C for 15 min. The reaction was then terminated by adding 50 μL of 2 M H_2_SO_4_. The absorbance of each well was then measured at 450 nm using a Multiskan Go Reader. The IgE-binding capacity was then measured using the formula:

(6) $$\mathrm{IgE}\;\text{binding capacity}\;\left(\%\right)=\frac{A_{0}-A}{A_{0}-A_{b}}\times 100$$

where A_0_, A and A_b_ are the absorbance of control β-LG, β-LG-EGCG conjugates and blank respectively.

### 2.12 Statistical analysis

Each sample was done in triplicate. The results are presented as mean ± standard deviation. Subsequently, moving forward, the value was found through one-way analysis of variance (ANOVA). The Waller-Duncan's multiple range test (Type I/Type II Error Ratio is 100) was used to evaluated the means at a significant level of *p* < 0.05. Structural schematic representations of β-LG were done in PyMOL (2.5.0), starting from the crystal structure PDB: 3NPO.

## 3 Results and discussion

### 3.1 SDS-PAGE analysis

**Fig. 1:**
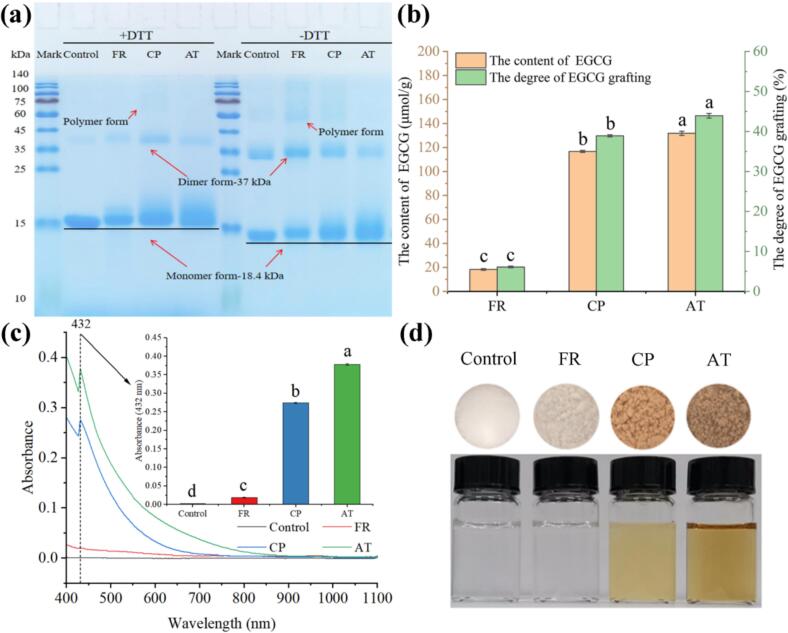
Characterization of β-LG-EGCG conjugates prepared with different method (FR, CP, and AT) and theirs browning degree. (a) SDS-PAGE analysis; (b) The content of EGCG in β-LG-EGCG conjugates; (c) Visual appearance of β-LG-EGCG conjugates powders and theirs solution; (d) Browning degree analysis. The degree of EGCG grafting means the percentage of the grafted EGCG in all added EGCG.

The molecular weight profile of β-LG after covalently grafted by EGCG using different methods (FR, CP, and AT) was monitored by reducing (+DTT) and non-reducing (-DTT) SDS-PAGE analysis. As depicted in Fig. 1a, the characteristic bands of β-LG were clearly observed around 18.4 kDa. Under reducing conditions (+DTT), compared with the control, slight upward migration and band smearing of β-LG were observed, implying the formation of β-LG-EGCG conjugates by FR, CP, and AT (He et al., 2023). In addition, the band tailing intensity increased progressively (FR < CP < AT), correlating with the degree of EGCG conjugation and indicating maximal polyphenol incorporation in AT conjugates. Moreover, dimeric and polymeric forms of β-LG were observed in all samples, with more pronounced features in CP. Due to disruption of disulfide bonds (-S-S-) under reducing conditions, dimeric and polymeric forms of β-LG were probably generated by covalent crosslinking between β-LG molecules via EGCG (Liu et al., 2024). Similar findings were reported by He et al. (2023), in which dimeric forms of peanut protein extract were generated by the crosslinking with chlorogenic acid and EGCG. On the other hand, similar electrophoretic patterns were observed under non-reducing conditions (-DTT), with all samples exhibiting intensified dimeric β-LG bands due to preserved disulfide crosslinking.

### 3.2 Content of EGCG in β-LG-EGCG conjugates

The Foline-Ciocalteu assay was employed to quantify the EGCG content in the covalent β-LG-EGCG conjugates. As shown in Fig. 1b, the EGCG grafting degree in β-LG-EGCG conjugates was significantly influenced by the conjugation method, with AT-treated samples showing the highest binding degree (131.79 ± 1.75 μmol/g, equivalent to 43.93 ± 0.58 % of grafting), followed by CP (116.73 ± 0.82 μmol/g, equivalent to 38.91 ± 0.27 % of grafting) and FR (18.29 ± 0.67 μmol/g, equivalent to 6.10 ± 0.22 % of grafting). These findings correlated well with the electrophoretic mobility patterns observed by SDS-PAGE (Fig. 1a). Wu et al. (2018) reported polyphenol contents of 34.2 ± 1.4 mg/g (equivalent to 74.67 μmol/g and 24.89 % of grafting) and 13.3 ± 0.9 mg/g (equivalent to 37.57 μmol/g and 12.52 % of grafting) in β-LG-EGCG and β-LG-chlorogenic acid conjugates formed by free-radical treatment method. Similarly, Man et al. (2024) used alkaline treatment method forming the β-LG-EGCG conjugate with an EGCG content of 75.97 μmol/g (equivalent to 69.59 % of grafting).

### 3.3 Degree of browning analysis

Browning is an important factor affecting the visual acceptability of food (Martin, Jacob-Dolan, Pham, Sjoblom, & Scheck, 2024). The differences in the browning degree of β-LG-EGCG covalent conjugates formed by different methods were determined. UV–Vis spectroscopy (400–1100 nm) revealed a characteristic absorption maximum at 432 nm for browning products across all conjugates (Fig. 1c). Among these β-LG-EGCG conjugates, the highest browning intensity, with an absorbance of 0.378 ± 0.002 was observed in AT. Subsequently, it was 0.274 ± 0.002 in CP. The minimum was 0.020 ± 0.001 in FR. These results were consistent with the visual appearance of the samples in Fig. 1d. Similar color-browning phenomena were observed in egg white protein -rosmarinic acid (Man et al., 2024); and soy protein isolate -proanthocyanidins conjugates (Pi et al., 2023) formed by the alkaline treatment method. The browning of covalent β-LG-EGCG conjugates may be attributed to the semi-quinone radicals, quinones and their polymers (brown products) generated by EGCG oxidation in protein-polyphenol covalent conjugation reactions (Yan et al., 2023). Notably, CP treatment achieved comparable EGCG conjugation efficiency (11.4 % lower than AT) while significantly reducing browning by 27.51 % (Fig. 1c), demonstrating its dual advantage as an efficient protein-polyphenol conjugation strategy that minimizes oxidative discoloration.

### 3.4 Lc-MS/MS

**Fig. 2:**
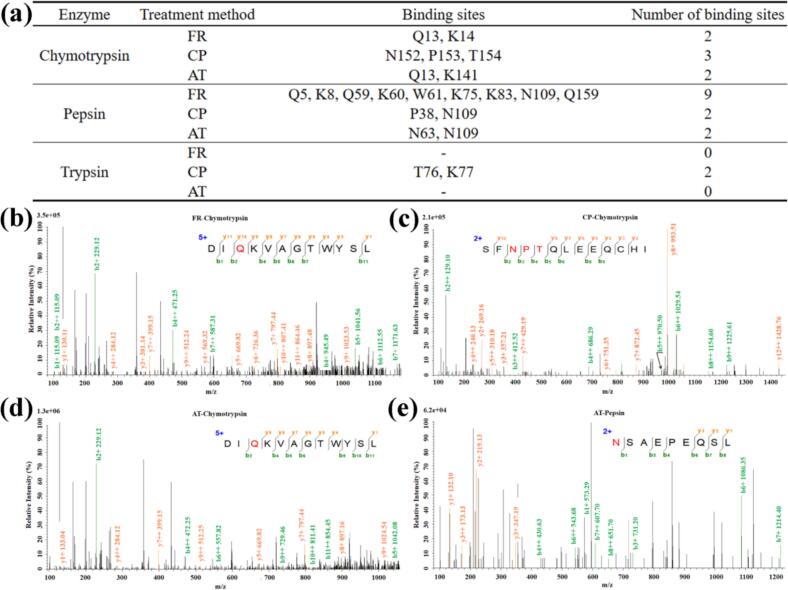
The covalent binding site of EGCG in β-LG-EGCG conjugates analyzed by LC-MS/MS. (a) The table of LC-MS/MS results; (b) Peptide of KGLDIQKVAGTWY in FR sample hydrolyzed by chymotrypsin; (c) Peptide of SFNPTQLEEQCHI in CP sample hydrolyzed by chymotrypsin; (d) Peptide of DIQKVAGTWYSL in AT sample hydrolyzed by chymotrypsin; (e) Peptide of NSAEPEQSL in AT sample hydrolyzed by pepsin. The red marked amino acids are the binding sites of EGCG on β-LG-EGCG conjugates. (For interpretation of the references to color in this figure legend, the reader is referred to the web version of this article.)

The variation in binding sites of covalent β-LG-EGCG conjugates formed by different methods was systematically identified using LC-MS/MS coupled with pre-hydrolysis by three enzymes (trypsin, pepsin, and chymotrypsin) (Fig. 2a). Typically, trypsin cleaves polypeptide chains at the C-terminal side of Arg or Lys residues (Sun et al., 2020), while pepsin targets aromatic residues (Phe, Tyr, Trp), and chymotrypsin cleaves at the C-terminal side of Phe, Tyr, and Trp, along with Leu and Met (Giansanti, Tsiatsiani, Low, & Heck, 2016a). Despite the widespread use of trypsin in protein-polyphenol binding site analysis (Unterhauser et al., 2023), trypsin was notably less effective in binding site analysis in the current study (Fig. 2a). In sum, a total of 11 binding sites (Q5, K8, Q13, K14, Q59, K60, W61, K75, K83, N109 and Q159) in the FR sample, 7 binding sites (P38, T76, K77, N109, N152, P153 and T153) in the CP sample, and 4 binding sites (Q13, N63, N109 and K141) in the AT sample were detected by LC-MS/MS, respectively. Notably, residue N109 was consistently identified as a primary EGCG-binding site in pepsin-hydrolyzed FR, CP, and AT samples, indicating its strong affinity for covalent conjugation. MS^2^ spectral analysis confirmed binding site localization (Fig. 2b and S Fig. 2a-n), exemplified by the Q13 site on peptide DIQKVAGTWYSL, where a mass shift between y_**10**_ and y_**9**_ ions matched the combined molecular weight of glutamine and EGCG. It is worth noting that some low-intensity y and b ions may be lost. Similarly, Zhou et al. (2020) found that EGCG was prone to bind with C, H, R and K in SPI-EGCG conjugates under the alkaline treatment method. Analogously, Sun et al. (2022) reported that the H, C and W were the most highly reactive amino acids involved in the egg white protein-EGCG, −chlorogenic acid, −caffeic acid, −catechin and -quercetin conjugates formed by free-radical treatment method. Overall, LC-MS/MS results confirmed the formation of β-LG-EGCG covalent conjugates, which is consistent with SDS-PAGE and EGCG content analysis.

**Fig. 3:**
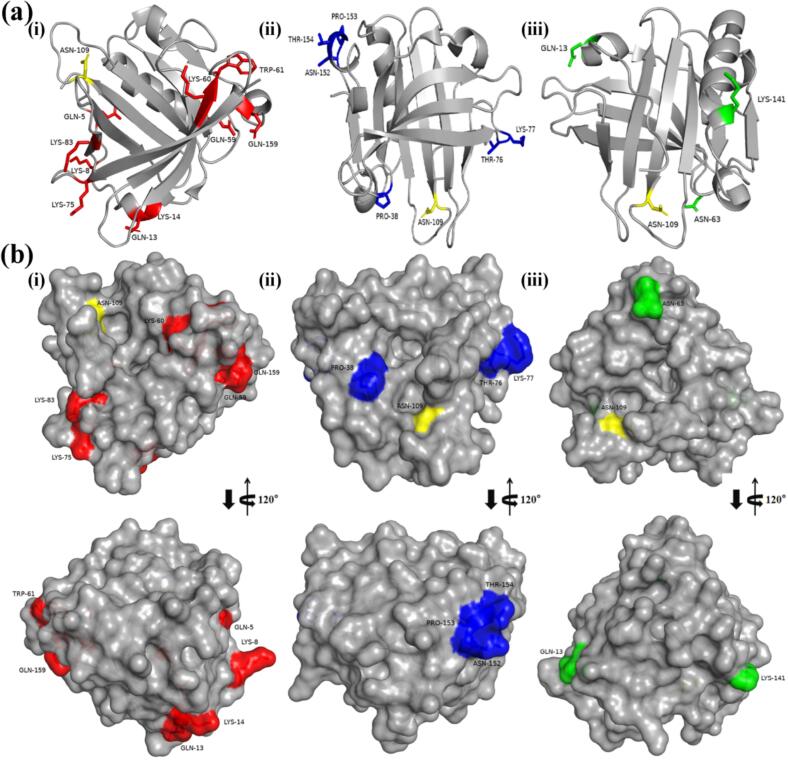
Schematic representation for the detected binding sites in β-LG (PDB ID: 3NPO). (a) Schematic representation for the binding sites in the secondary structure β-LG: (i) FR sample; (ii) CP sample; (iii) AT sample. (b) Schematic representation for the binding sites on β-LG surface: (i) FR sample; (ii) CP sample; (iii) AT sample.

**Fig. 4:**
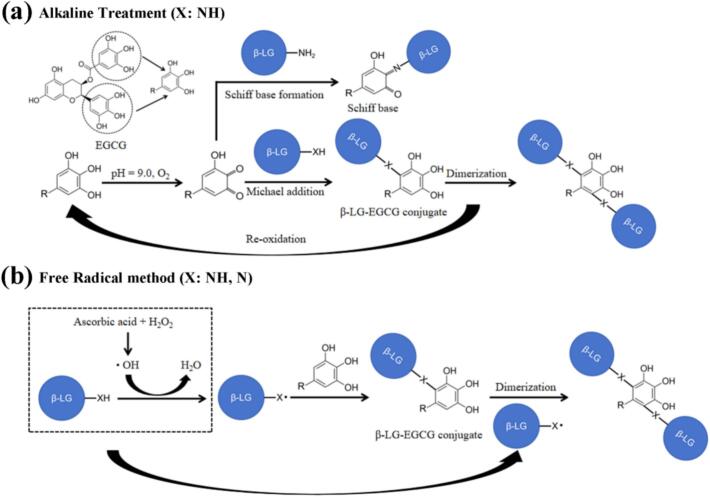
A schematic of type of bonds between β-LG and EGCG formed by alkaline treatment and free radical method.

The binding sites identified by LC-MS/MS exhibited the following preferences: (1) For FR and AT, EGCG tended to bind to amino acids with side chains containing -NH_2_ and -NH (Q, K, N, W), indicating that -NH_2_ and -NH groups are crucial for the formation of β-LG-EGCG covalent conjugates formed by the two methods; (2) For FR, CP and AT, except for W61, P38, and P153, all other binding sites are hydrophilic amino acids, suggesting that hydrophilic amino acids were more likely to be bound by EGCG; (3) For FR, CP and AT, the binding sites were all located on the surface of β-LG (Fig. 3b), indicating that spatial accessibility is crucial for EGCG to bind to β-LG. Moreover, it is worth noting that compared to the FR and AT, the β-LG-EGCG conjugate formed by CP method enriched the variety of amino acid for EGCG binding. In addition to amino acids containing -NH_2_ and -NH groups in the side chain, the CP method can also promote the binding of EGCG to amino acids containing -OH (T76, T154) and cyclic-alkyl groups (P38, P153) in the side chain. Additionally, the LC-MS/MS results directly inform the potential chemical bonding between β-LG and EGCG. The consistent identification of binding at residues with nucleophilic side chains (-NH₂, -NH, -OH) aligns with established covalent conjugation mechanisms. Specifically, binding to Lysine (K) and Tryptophan (W) suggests the formation of Michael addition adducts (C—N) or Schiff bases (C=N), where the nucleophilic amino group attacks the electrophilic quinone of oxidized EGCG (Fig. 4) (X. Yan et al., 2023). The unique binding to Threonine (T) containing a hydroxyl group (-OH) in CP conjugates further indicates the potential for ether bond (C-O-C) formation or other radical-mediated coupling pathways, highlighting the distinct reactivity induced by CP treatment. Further research should be done to elucidate the specific chemical bonding to linkage the protein and polyphenol in conjugated induced by CP.

### 3.5 Structural alteration analysis

#### 3.5.1 Secondary structure

**Fig. 5:**
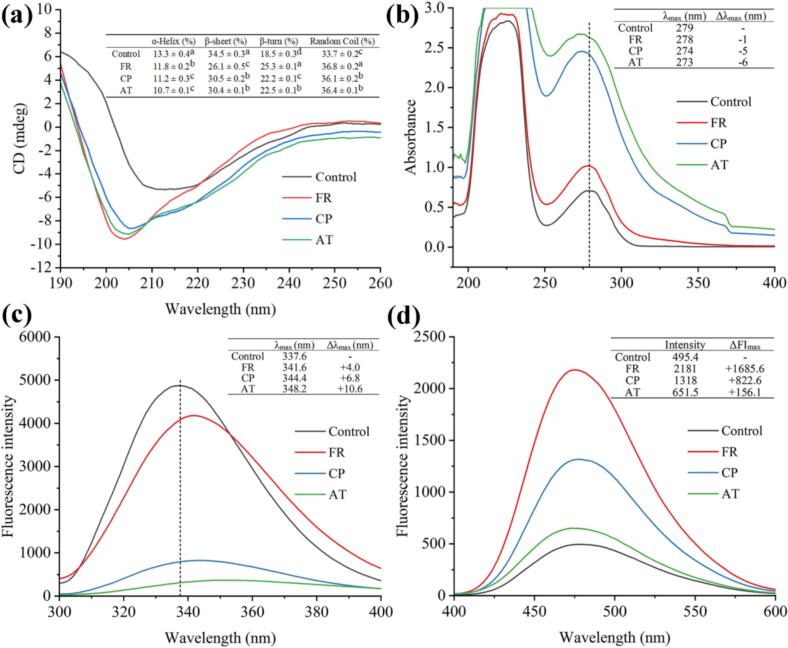
The results of multispectral analysis of β-LG-EGCG conjugates prepared with different method (FR, CP, and AT). (a) CD analysis; (b) UV–vis spectrum analysis; (c) Intrinsic fluorescence analysis; (d) Surface hydrophobicity analysis. The Δλ_max_ and ΔFI_max_ are compared to the control. The ‘+’ means increased intensity of FI_max_ and red-shift of λ_max_, respectively. And the ‘-’ means decreased intensity of FI_max_ and blue-shift of λ_max_, respectively. (For interpretation of the references to color in this figure legend, the reader is referred to the web version of this article.)

Alterations in the secondary structure of β-LG after covalent grafting with EGCG using different methods were monitored by CD. As depicted in Fig. 5a, clear changes in the CD spectra were observed in β-LG-EGCG conjugates compared with the control, indicating variations in the secondary structure proportions of β-LG after covalently conjugated by EGCG. For the control, the secondary structure comprised 13.3 ± 0.4 % α-helix, 34.5 ± 0.3 % β-sheet, 18.5 ± 0.3 % β-turn, and 33.7 ± 0.2 % random coil, which is consistent with our previous study (Lv et al., 2024). Compared to the control, the secondary structure of β-LG exhibited the common trends after covalent grafting with EGCG using different methods, namely a marked reduction in ordered structures (α-helix and β-sheet) accompanied by elevation in flexible regions (β-turn and random coil) (Yan et al., 2025). Specifically, the α-helix content declined from 13.3 ± 0.4 % (control) to 10.7 ± 0.1 % (AT), while β-sheet content decreased from 34.5 ± 0.3 % to 26.1 ± 0.5 % (FR). Concurrently, the β-turn and random coil contents rose from 18.5 ± 0.3 % and 33.7 ± 0.2 % to 25.3 ± 0.1 % (FR) and 36.8 ± 0.2 % (FR), respectively. These changes imply that covalent conjugation with EGCG might disrupted β-LG's native structure, diminishing its compactness and promoting partial unfolding, which is consistent with our previous research (Lv et al., 2024). Similar results were obtained by Yan et al. (2025), who reported increased random coil content and decreased β-sheet and α-helix content in soy protein isolate-soybean isoflavone conjugates formed by alkaline treatment. Analogously, Liu et al. (2025) reported that α-helix of pea protein isolated decreased by 12.5 % and random coli increased by 4 % after covalently conjugated with gallic acid via free-radical treatment.

#### 3.5.2 Tertiary structure and micro-environment

UV–vis spectroscopy, intrinsic fluorescence spectroscopy and surface hydrophobicity analyses were employed to evaluate tertiary structure and micro-environment changes of β-LG upon conjugation with EGCG. The UV–vis absorption spectrum results are depicted in Fig. 5b. The control sample exhibited a characteristic absorption peak at 279 nm (around 280 nm), arising from the π-π* transitions of Tyr, Trp and Phe (Wang et al., 2025). A maximum absorption peak wavelength shift (Δλ_max_) of β-LG with −1 (FR), −5 (CP) and − 6 (AT) nm, respectively, was observed, accompanied by the enhanced absorption intensity, which positively correlated with the EGCG grafting degree in the conjugates. The stability of the α-helix and β-sheet structures is primarily maintained by hydrogen bonding. Covalent conjugation with EGCG likely perturbed this stabilizing network, leading to a loss of α-helix and β-sheet content, which indicates the partial unfolding of β-LG (Chen, Chen, Jiang, Wang, & Zhang, 2025). Hydrophobic aromatic residues (Tyr and Trp) are buried with in β-LG's structure. Increased solvent (water) exposure may increase the hydrophilicity of their microenvironment, resulting in increased intensity and a blue-shift of λ_max_ (Chen et al., 2025). Therefore, the above results indicate that after covalent binding with EGCG by different methods, the micro-environment hydrophilicity of Tyr and Trp increased, suggesting that the β-LG spherical structure unfolded, which is consistent with the results of secondary structure changes.

Changes in fluorescence intensity of β-LG after conjugation with EGCG are shown in Fig. 5c. A maximum fluorescence intensity (FI_max_) of 4875 at 337.6 nm was observed in the control. An obvious Δλ_max_ with +4.0 (FR), +6.8 (CP) and + 10.6 (AT) nm, respectively, was detected, aligned with a gradual decrease in intensity of FI_max_. These findings indicate that EGCG grafting perturbed the conformation of β-LG, resulting in unfolding of the native structure and a more polar microenvironment around the hydrophobic fluorophore (Trp) buried in native β-LG (Wang et al., 2025), which is consistent with the CD and UV–vis results. Similar results were obtained by J. Liu et al. (2023), who reported that following conjugation with chlorogenic acid by free-radical treatment method, FI_max_ decreased and λ_max_ exhibited a red-shift in β-LG.

Changes in surface hydrophobicity of β-LG after conjugated with EGCG were evaluated (Fig. 5d). Compared with the control, a maximum fluorescence intensity shift (ΔFI_max_) with +1685.6 (FR), +822.6 (CP) and + 156.1 (AT), respectively, was observed upon conjugation with EGCG, implying increased surface hydrophobicity of β-LG-EGCG conjugates. In addition, a negative correlation was observed between FI_max_ and the covalent grafting degree of EGCG in the conjugates, with FI_max_ progressively diminishing as more EGCG was grafted. Similar results were found in covalent β-LG-ferulic acid conjugates (Xue et al., 2023), and soy protein isolate-gallic acid, −caffeic acid and -tannic acid (Pi et al., 2023). Two factors underlie this phenomenon: (1) partial unfolding of β-LG structure induced by covalent grafting of EGCG, as corroborated by CD, UV–vis, and intrinsic fluorescence analyses; these structural rearrangements resulted in exposure of buried hydrophobic residues on the β-LG surface, transiently amplifying hydrophobicity; (2) bound EGCG sterically shielded hydrophobic regions, limiting ANS accessibility and resulting in fewer detectable hydrophobic sites, thereby reducing surface hydrophobicity. Overall, the tertiary structure and micro-environment analyses indicated that covalent grafting with EGCG caused unfolding of β-LG's conformation and exposure of buried hydrophobic groups.

### 3.6 Water contact angle

**Fig. 6:**
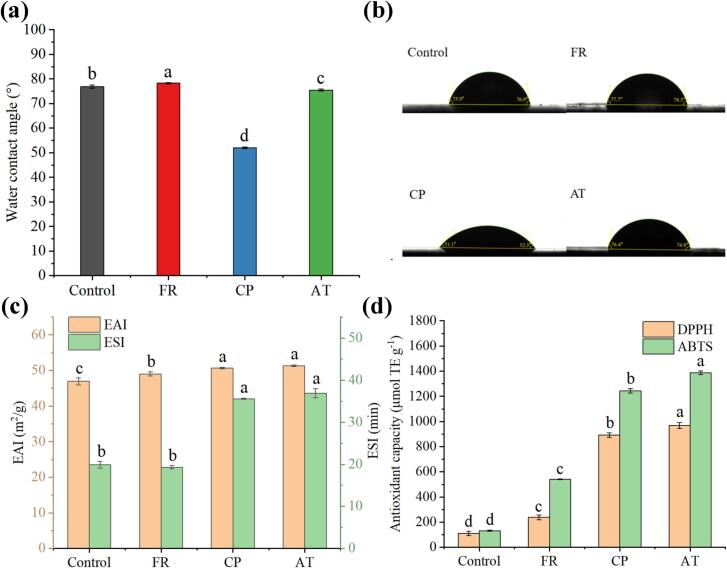
Results of wettability and functionalities analysis of β-LG-EGCG conjugates prepared with different method (FR, CP, and AT). (a) WCA; (b) The visual result of WCA; (c) Emulsifying property analysis; (d) Antioxidant capacity analysis.

The wettability of β-LG in FR, CP, and AT samples was evaluated by water contact angle (WCA) analysis. A WCA < 90° indicates hydrophilic surfaces (lower angles = higher hydrophilicity), whereas a WCA > 90° indicates hydrophobic surfaces (larger angles = greater hydrophobicity) (Wang et al., 2024). As illustrated in Fig. 6a and b, the WCA of the control was 76.9 ± 0.6°. After covalent grafting with EGCG, the WCA of FR slightly increased from 76.9 ± 0.6° (Control) to 78.3 ± 0.2°, indicating decreased in hydrophilicity of β-LG. In addition, the WCA of the CP and AT samples decreased to 52.0 ± 0.3°and 75.5 ± 0.4°, respectively. The discrepancy between the surface hydrophobicity analysis (Section 3.5.2) and WCA measurements may be explained as follows: (1) covalently bound EGCG introduces additional hydrophilic hydroxyl (-OH) groups onto β-LG surface, with the number of -OH groups increasing with EGCG content, thereby enhancing surface hydrophilicity; (2) compared to AT, CP exhibited a significantly lower WCA, likely due to the introduction of additional hydrophilic groups by CP treatment (Zhu, Cheng, Han, & Han, 2023). Consistent with our findings, Wang et al. (2024) reported that alkaline-mediated conjugation with rosmarinic acid increased the WCA of β-LG from 66.7 ± 0.5° to 73.8 ± 0.3°. In summary, the surface wettability of β-LG was modulated by the degree of EGCG covalent conjugation. Notably, at equivalent conjugation degrees, CP-induced β-LG-EGCG covalent conjugates exhibited a significantly reduced WCA compared with FR and AT methods.

### 3.7 Functionalities analysis

#### 3.7.1 Emulsifying properties

The emulsifying properties of β-LG and its EGCG conjugates were evaluated using the emulsifying activity index (EAI) and the emulsion stability index (ESI) measurements. As illustrated in Fig. 6c, native β-LG exhibited considerable emulsifying capacity (EAI = 46.95 ± 1.02 m^2^/g) but limited stability (ESI = 19.91 ± 0.80 min). EGCG conjugation significantly enhanced both of indices, with maximal values reaching 51.36 ± 0.20 m^2^/g (EAI) and 36.92 ± 1.09 min (ESI) in AT. The CP-induced conjugates showed comparable improvements, with EAI of 50.7 ± 0.23 m^2^/g and ESI of 35.59 ± 0.16 min. Notably, a clear concentration-dependent enhancement in the emulsifying properties of β-LG with increasing EGCG incorporation was observed, suggesting that covalent grafting of EGCG effectively improved the protein's interfacial properties. The enhanced emulsifying properties, particularly the emulsion stability, of β-LG upon conjugation with EGCG can be attributed to its structural unfolding and the resultant amphiphilic character. The covalent grafting induces partial unfolding of β-LG, exposing hydrophobic regions that facilitate its adsorption to the oil-water interface. Concurrently, the hydrophilic EGCG moieties extend into the aqueous phase, providing strong steric repulsion between oil droplets. This synergy of improved surface activity and effective steric stabilization promotes the formation of a robust interfacial film, thereby significantly enhancing emulsifying performance of β-LG-EGCG conjugates (Wang et al., 2025). Consistent with our findings, Man et al. (2024) reported that alkaline-mediated conjugation with caffeic acid, chlorogenic acid, and EGCG significantly improved the emulsifying properties of β-LG, with β-LG-EGCG demonstrating the most pronounced enhancement (56.17 % increase in EAI and 44.39 % in ESI). Similarly, Wang et al. (2025) observed improved emulsification performance in alkaline-treated egg white protein-rosmarinic acid conjugates.

#### 3.7.2 Antioxidant capacity

The radical scavenging capacities of β-LG and its conjugates were evaluated using DPPH and ABTS assays. As depicted in the Fig. 6d, covalent EGCG conjugation markedly enhanced β-LG's antioxidant capacity, with the following order: AT > CP > FR > Control. Notably, the antioxidant capacity of β-LG-EGCG conjugates was positively correlated with EGCG content, reaching maxima in AT with 969.24 ± 24.14 μmol TE g^−1^ (DPPH) and 1386.64 ± 13.62 μmol TE g^−1^ (ABTS), which were approximately 9-fold and 10-fold higher than those of the control, respectively. For CP-induced β-LG-EGCG conjugates, the radical scavenging capacities were 892.76 ± 17.06 μmol TE g^−1^ (DPPH) and 1243.98 ± 17.12 μmol TE g^−1^ (ABTS). Similarly， Liu et al. (2024) demonstrated that CP-induced ovalbumin-gallic acid conjugates exhibited dose-dependent enhancements in both DPPH and ABTS radical scavenging capacities with increasing grafted degree of gallic acid. In conclusion, covalent EGCG conjugation represents an effective strategy for simultaneously enhancing the antioxidant and emulsifying properties of β-LG. Notably, CP-mediated conjugation offers distinct advantages over conventional FR and AT methods: (1) it significantly improves β-LG's emulsification performance while minimizing polyphenol oxidation, thereby better preserving EGCG's intrinsic antioxidant capacity; and (2) it reduces undesirable color changes, demonstrating superior potential for food-grade protein modification. These findings underscore the practical utility of CP technology in developing functional protein-polyphenol conjugates for food applications.

### 3.8 In vitro digestibility analysis

**Fig. 7:**
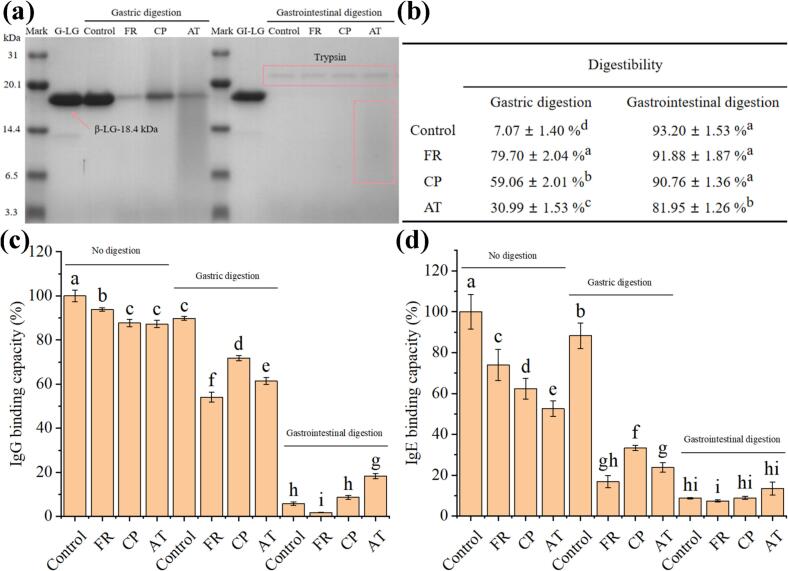
The digestibility and change in antigenicity during In vitro simulated gastrointestinal digestion of β-LG-EGCG conjugates prepared with different method (FR, CP, and AT). (a) Tricine-SDS-PAGE analysis; (b) Digestibility analyzed by TCA. (c) IgG binding capacity; (d) IgE binding capacity.

In vitro simulated gastrointestinal digestion analysis was conducted to evaluate the digestibility of β-LG and covalent β-LG-EGCG conjugates. Distinct digestibility patterns between native β-LG and its EGCG conjugates are displayed in Fig. 7a. Results of Tricine SDS-PAGE analysis indicated that native β-LG maintained its characteristic band (∼18.4 kDa) throughout gastric digestion, consistent with its known pepsin resistance (Cao & Xiong, 2017). This stability arises from: (1) preservation of the β-barrel structure at gastric pH (Jia, Zhu, Wang, Peng, & Shi, 2022), and (2) burial of pepsin cleavage sites (Y, F, W) within its hydrophobic cores (Giansanti, Tsiatsiani, Low, & Heck, 2016b). In contrast, all conjugates showed markedly reduced band intensities, indicating enhanced gastric digestibility upon EGCG conjugation. This improvement likely stems from structural unfolding (Fig. 5) that exposes hydrophobic residues and cleavage sites to pepsin (L. Li et al., 2023). Among conjugates, FR-induced conjugates exhibited the most complete digestion (minimal residual peptides), while AT samples showed intermediate digestion (6.5–14.4 kDa fragments). These findings align with reported pepsin susceptibility enhancements in alkaline-induced soybean 11 s globulin-EGCG conjugates (Li et al., 2023). As depicted in Fig. 7a, compared to the undigested sample (GI-LG), complete disappearance of the characteristic β-LG band after subsequent gastrointestinal digestion confirmed extensive proteolysis of β-LG and its conjugates. Notably, while FR- and CP-induced conjugates exhibited complete digestion comparable to the control, AT conjugates retained residual peptide fragments (6.5–14.4 kDa), suggesting marginally reduced digestibility relative to the other conjugates.

TCA precipitation method was further used to evaluate the degree of digestion degree of β-LG and its conjugates. As shown in Fig. 7b, the gastric digestion rates of samples were 7.07 ± 1.40 % (Control), 79.70 ± 2.04 % (FR), 59.06 ± 2.01 % (CP), and 30.99 ± 1.53 % (AT), respectively, which are consistent with the Tricine SDS-PAGE results, indicating increased gastric digestion of the β-LG-EGCG conjugate. Of note, the digestibility of conjugates gradually decreased as the EGCG binding amount increased. This phenomenon may be attributed to the fact that, although covalent conjugation with EGCG induced structural unfolding of β-LG, thereby increasing pepsin accessibility to hydrolysis sites and enhancing gastric digestibility, the bound EGCG molecules simultaneously exerted steric-shielding effects that partially obstructed pepsin cleavage sites, resulting in an overall reduction in proteolytic efficiency (J. Yan et al., 2025). In addition, the gastrointestinal digestibility rates were 93.20 ± 1.53 % (Control), 91.88 ± 1.87 % (FR), 90.76 ± 1.36 % (CP), and 81.95 ± 1.26 % (AT), respectively. These results indicate that β-LG has high gastrointestinal digestibility but is resistant to gastric digestion (Jia et al., 2022). Compared to the control, an obvious decrease in the gastrointestinal digestion was discerned in AT-induced β-LG-EGCG conjugates, which may be attributed to covalent EGCG grafting partly blocking trypsin hydrolysis sites (Dai et al., 2023). Consistent with our findings, Yan et al. (2025) reported reduced gastrointestinal digestibility in alkaline-induced soy protein isolate-isoflavone conjugates. In summary, CP treatment effectively facilitated β-LG-EGCG covalent conjugation while simultaneously enhancing the gastrointestinal digestibility of β-LG.

### 3.9 Antigenicity analysis of β-LG-EGCG conjugates and its digested samples

The immunoglobulin (IgG/IgE) binding capacities of β-LG and its conjugates before/after digestion were evaluated. As depicted in Fig. 7c and d, the IgG and IgE binding abilities of β-LG-EGCG gradually decrease to 93.83 ± 0.71 % (FR), 87.70 ± 1.73 % (CP), 87.23 ± 1.64 % (AT) and 73.97 ± 7.67 % (FR), 62.36 ± 5.06 % (CP), 52.62 ± 3.74 % (AT), respectively, which was positively correlated with the EGCG grafting degree. The masking/disrupting effects on the β-LG's linear and conformational epitopes of IgG and IgE by EGCG may account for the reduction in immunoglobulin binding capacity of β-LG-EGCG (C. Liu et al., 2025a). Similarly, the antigenicity of β-LG-ferulic acid conjugates formed by the alkaline treatment method decreased as the grafted ferulic acid content increased (Xue et al., 2023). After gastric digestion, the IgG binding abilities were 89.75 ± 0.88 % (Control), 54.11 ± 2.27 % (FR), 71.83 ± 1.14 % (CP), and 61.40 ± 1.58 % (AT), respectively, while the IgE binding abilities were 88.22 ± 6.23 % (Control), 16.84 ± 2.92 % (FR), 33.33 ± 1.34 % (CP), and 23.74 ± 2.31 % (AT), respectively. Notably, the FR sample exhibited the most pronounced reduction in IgG/IgE binding activity. These results may be attributed to the high digestibility of the FR sample in the gastric phase (Fig. 7a and b). In addition, the binding ability of IgG and IgE showed the following trend: Control > CP > AT > FR, which was consistent with the intensity trend of the β-LG characteristic bands in the gastric phase of the samples (Fig. 7a).

The binding ability of IgG and IgE in all samples was substantially reduced after gastrointestinal digestion (Fig. 7c and d). The IgG and IgE binding ability of all samples decreased to below 9 %, except for AT-induced β-LG-EGCG conjugates. Among them, the lowest IgG and IgE binding abilities were obtained in FR, with values of 1.80 ± 0.17 % (IgG) and 7.41 ± 0.58 % (IgE), subsequent was in CP, with the amount of 8.66 ± 1.82 % (IgG) and 8.92 ± 0.77 % (IgE). These finding implied that the elimination of immunoglobulin binding capacities of β-LG and its conjugates was directly related to the gastrointestinal digestibility of samples. Similar results were found by Li et al. (2023) and Wang et al. (2021). In summary, CP treatment not only facilitates rapid and efficient covalent conjugation between β-LG and EGCG, but also exhibits lower antigenicity during gastrointestinal digestion compared to AT at an equivalent EGCG conjugation degree.

In conclusion, the collective findings of this study underscore the significant potential of CP as a novel processing strategy for developing low-allergenic milk protein ingredients. The covalent conjugation induced by CP effectively masked immunoglobulin epitopes on β-LG, as evidenced by the reduced IgG/IgE binding capacity. This reduction in antigenicity, coupled with the observed enhancement in gastric digestibility, suggests that CP treatment can promote the breakdown of β-LG into less immunogenic peptides during digestion. Furthermore, the concurrent improvements in emulsifying and antioxidant properties ensured that the functional value of the protein is not only preserved but enhanced. Therefore, the CP technique presents a compelling, efficient, and chemical-free alternative to conventional methods for fabricating β-LG-polyphenol conjugates with superior safety and functionality, holding great promise for their application in hypoallergenic infant formulas and other specialized dairy products.

## 4 Conclusion

This study systematically compared the structural, functional, and immunological properties of β-LG-EGCG conjugates prepared via CP, AT, and FR methods. Key findings demonstrate that CP treatment achieved near-equivalent EGCG grafting efficiency (116.73 ± 0.82 μmol/g, equivalent to 38.91 ± 0.27 % of grafting) to AT (131.79 ± 1.75 μmol/g, equivalent to 43.93 ± 0.58 % of grafting) after 45 s of CP treatment, while significantly outperforming FR (18.29 ± 0.67 μmol/g, equivalent to 6.10 ± 0.22 % of grafting). Notably, CP uniquely expanded EGCG-binding sites to include hydroxyl- and cyclic-alkyl-containing residues. Structurally, covalent grafting with EGCG caused unfolding of β-LG's structure unfolding and exposure of buried hydrophobic groups. CP mitigated oxidative browning by 27.51 % relative to AT, thereby helping to preserve EGCG's antioxidant capacity. Functionally, CP conjugates rivaled AT in emulsifying properties but demonstrated superior gastric digestibility and lower post-digestion antigenicity. These results underscore CP as a green, efficient alternative to conventional methods, balancing high conjugation yield with minimal oxidative degradation and enhanced functionality. Future research should explore CP's scalability and in vivo allergenicity to advance its application in hypoallergenic functional foods.

## CRediT authorship contribution statement

**Zhi-Wei Liu:** Writing – original draft, Methodology, Formal analysis, Data curation, Conceptualization. **Jun Lv:** Writing – review & editing, Formal analysis, Conceptualization. **Chang Liu:** Writing – review & editing. **Jun-Hu Cheng:** Writing – review & editing. **Najla AlMasoud:** Writing – review & editing, Conceptualization. **Rana Muhammad Aadil:** Writing – review & editing, Supervision, Conceptualization. **Xiu-Bin Liu:** Writing – review & editing, Supervision, Methodology, Funding acquisition, Conceptualization.

## Declaration of competing interest

The authors declare that they have no known competing financial interests or personal relationships that could have appeared to influence the work reported in this paper.

## Data Availability

Data will be made available on request.

## Appendix A Supplementary data

Supplementary material Image 1
