# Supplementary material for: Characterization of cold plasma-induced covalent epigallocatechin gallate conjugates of β-lactoglobulin: a comparison with free-radical and alkaline treatments
Source: Food Chem X. 2025 Dec 1;32:103330. doi: 10.1016/j.fochx.2025.103330 (PMC12719203; doi:10.1016/j.fochx.2025.103330)
Supplement: Supplementary file 1 — Supplementary material [file mmc1.docx]

**Supplementary materials**

S Table 1 Specific lgE levels (kU/L) of 14 patients against cow’s milk.

| Patient | Sex | Age (years) | IgE levels (kU/L) |
| --- | --- | --- | --- |
| 1 | Male | 3 | 4.06 |
| 2 | Female | 3 | 1.75 |
| 3 | Female | 3 | 19.04 |
| 4 | Male | 0.8 | 1.63 |
| 5 | Male | 9 | 3.91 |
| 6 | Female | 4 | 4.38 |
| 7 | Male | 3 | 4.85 |
| 8 | Female | 1 | 1.48 |
| 9 | Female | 3 | 2.22 |
| 10 | Female | 18 | 2.45 |
| 11 | Female | 3 | 5.57 |
| 12 | Female | 33 | 1.32 |
| 13 | Female | 6 | 1.15 |
| 14 | Female | 47 | 1.81 |

S Table 2 The polyphenols’ content standard curves.

| Polyphenols | Standard curves (R^2^ > 0.999) |
| --- | --- |
| EGCG | Y = 10.635X + 0.0063 |

Where X indicates the polyphenols content in mg/mL, and Y denotes the Abs measured at 760 nm.

S Table 3 The Trolox calibration curve.

| Free radical | Measurement wavelength | Standard curves (R^2^ > 0.999) |
| --- | --- | --- |
| DPPH | 517 nm | Y = 26.041X + 0.0016 |
| ABTS | 734 nm | Y = 38.721X - 0.008 |

Where X indicates the equivalent concentration of Trolox in mg/mL, and Y denotes scavenging rate.

S Table 4 The detail table of LC-MS/MS results

| **Treatment methods** | **Modified sequence** | **Mass (Da)** | **Binding site (s)** | **The type of side chains of binding amino acids** | | | |
| --- | --- | --- | --- | --- | --- | --- | --- |
|  |  |  |  | **Containing -NH_2_ group** | **Containing -NH group** | **Containing**  **-OH group** | **Containing  cyclic-alkyl group** |
| **FR** | **Q**TMKGLDIQ-  -KVAGTW | **2132.9612** | **Q5** | **Q5** | - | - | - |
|  | **LIVTQTM**KG | **1447.8127** | **K8** | **K8** | - | - | - |
|  | **DI**QKVAGTWYSL | **1837.7934** | **Q13** | **Q13** | - | - | - |
|  | **KGLDIQ**KVAGTWY | **1935.9627** | **K14** | **K14** | - | - | - |
|  | **LEILL**QKW | **2415.9619** | **Q59, K60, W61** | **Q59, K60** | **W61** | - | - |
|  | **KIIAE**KTKI-  -PAVFKIDALNE | **2698.3993** | **K75** | **K75** | - | - | - |
|  | **K**TKIPAVFKI | **1601.8229** | **K75** | **K75** | - | - | - |
|  | **K**IDALNENKV-  -LVLDTDYKKYL | **2952.4532** | **K83** | **K83** | - | - | - |
|  | **N**SAEPEQSL | **1431.5202** | **N109** | **N109** | - | - | - |
|  | **FNPTQLEE**Q | **1562.5937** | **Q159** | **Q159** | - | - | - |
| **CP** | **DAQSA**PLRV | **1413.5936** | **P38** | - | - | - | **P38** |
|  | **TK**IPAVFK | **1782.7076** | **T76, K77** | **K77** | - | **T76** | - |
|  | **N**SAEPEQSL | **1431.5202** | **N109** | **N109** | - | - | - |
|  | **N**SAEPEQS | **1318.4361** | **N109** | **N109** | - | - | - |
|  | **SF**NPTQLEEQCHI | **2975.9691** | **N152, P153, T154** | **N152** | - | **T154** | **P153** |
| **AT** | **DI**QKVAGTWYSL | **1837.7934** | **Q13** | **Q13** | - | - | - |
|  | **PEGDLEILLQK**-  -**WE**NGE | **2327.0005** | **N63** | **N63** | - | - | - |
|  | **N**SAEPEQSL | **1431.5202** | **N109** | **N109** | - | - | - |
|  | **DKAL**KAL | **1215.5547** | **K141** | **K141** | - | - | - |

|  |
| --- |


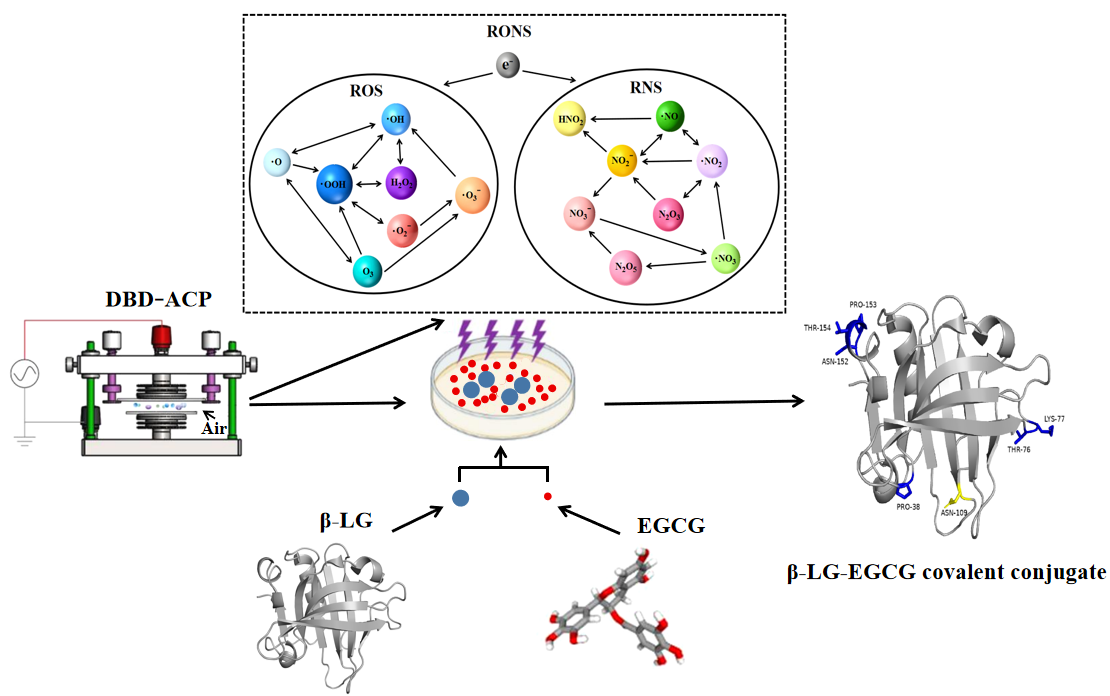


S Fig. 1 Graphical abstract illustrating the mechanism of CP in the formation of the EGCG-β-LG conjugate.


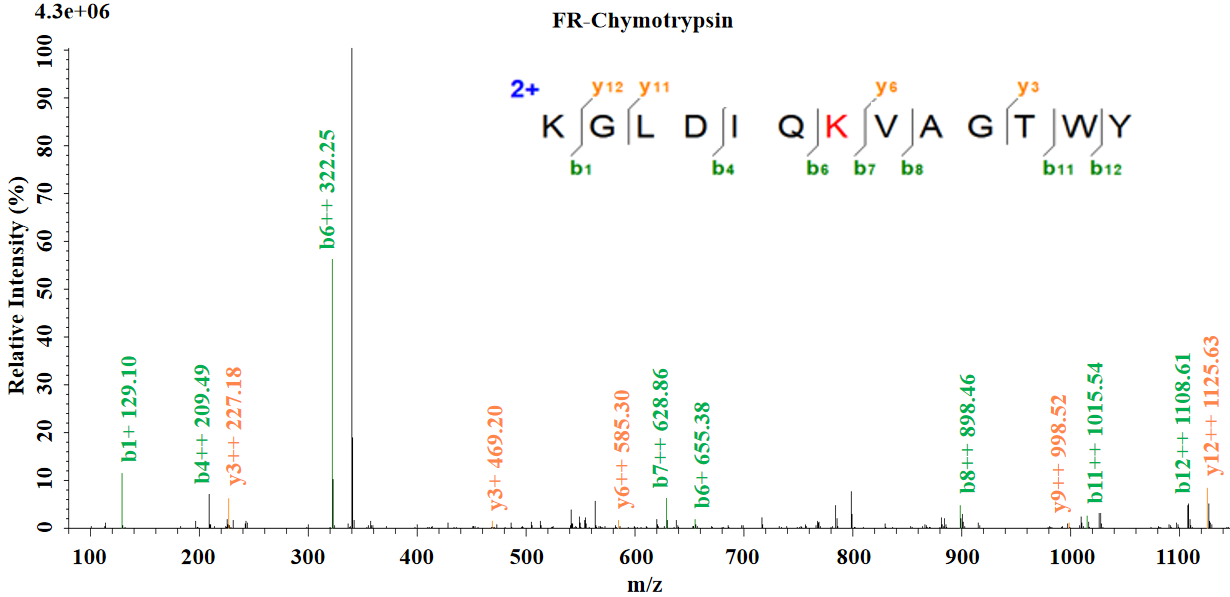


S Fig. 2a Peptide of KGLDIQKVAGTWY in FR sample [hydrolyze](file:///C:/Program%20Files/baidu-translate-client/resources/app.asar/app.html#/#)d by chymotrypsin. The red marked amino acids are the binding sites of EGCG on β-LG-EGCG conjugates.


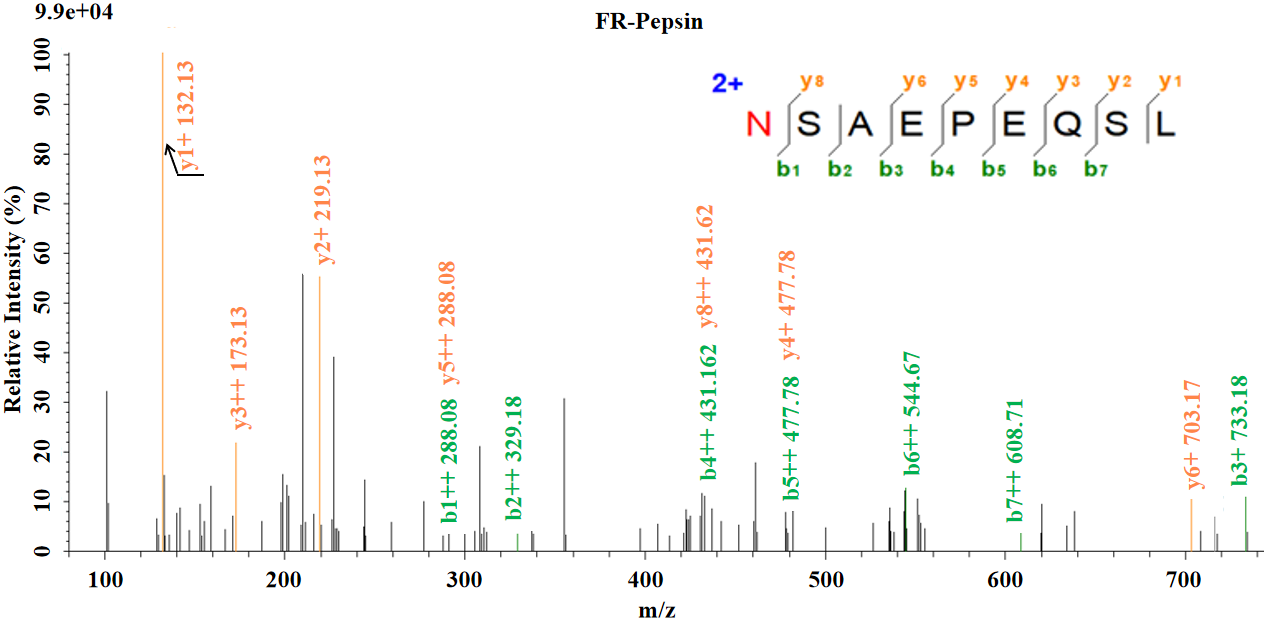


S Fig. 2b Peptide of NSAEPEQSL in FR sample [hydrolyze](file:///C:/Program%20Files/baidu-translate-client/resources/app.asar/app.html#/#)d by pepsin.


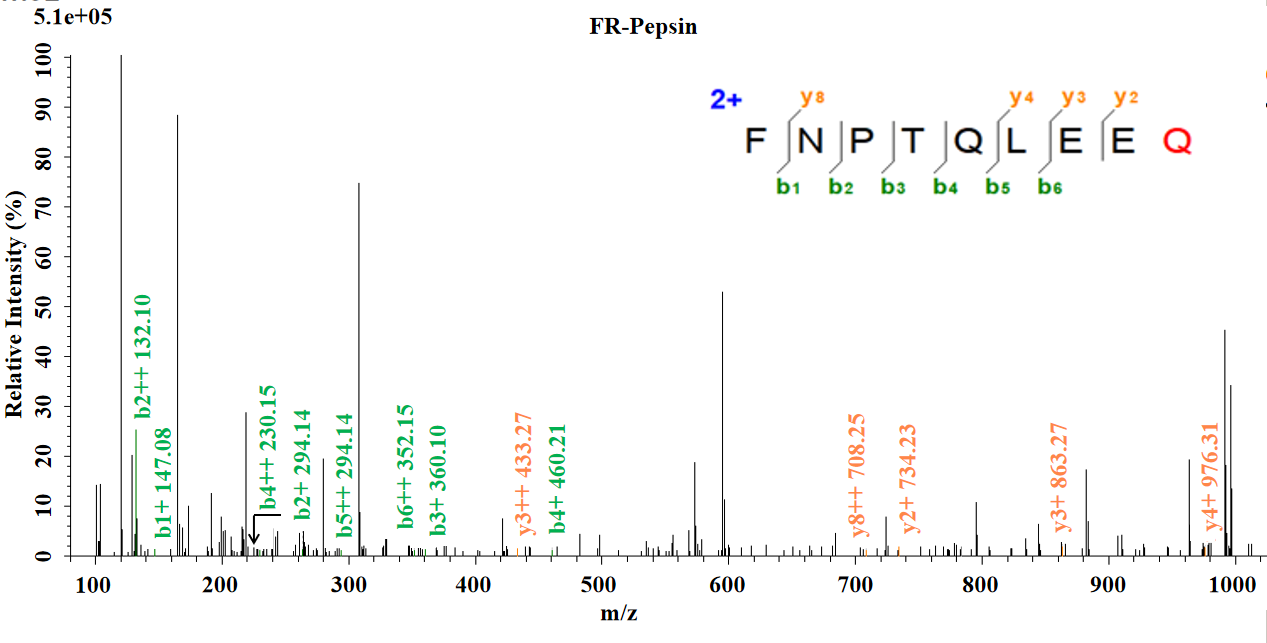


S Fig. 2c Peptide of FNPTQLEEQ in FR sample [hydrolyze](file:///C:/Program%20Files/baidu-translate-client/resources/app.asar/app.html#/#)d by pepsin


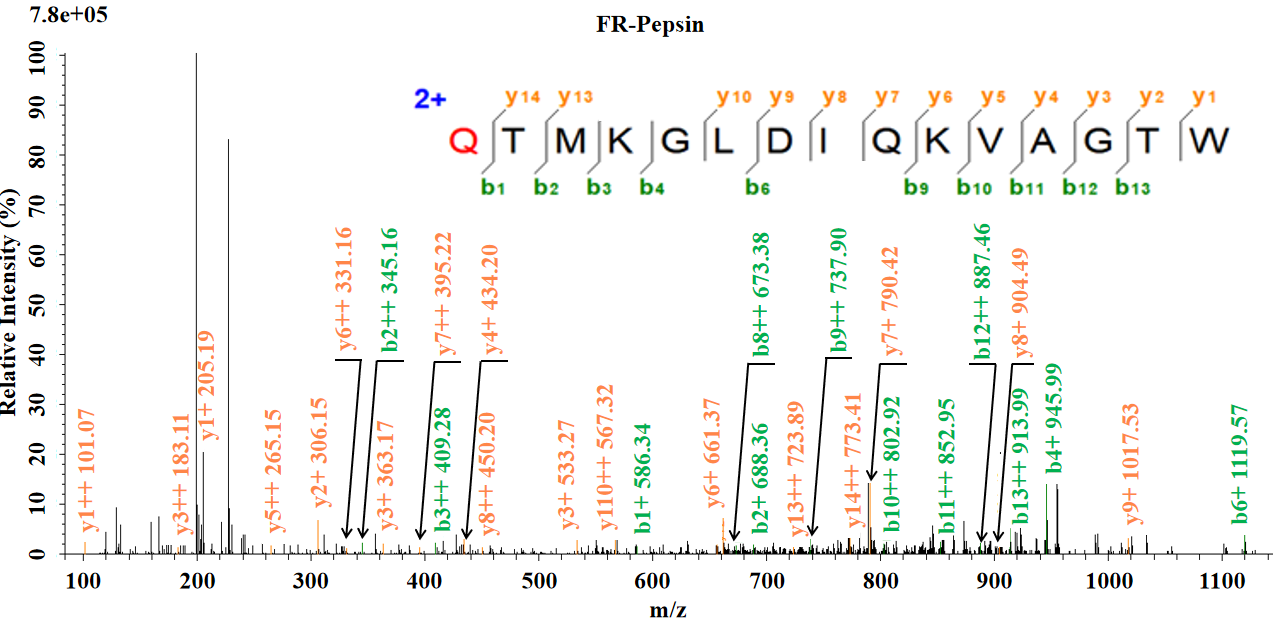


S Fig. 4d Peptide of QTMKGLDIQKVAGTW in FR sample [hydrolyze](file:///C:/Program%20Files/baidu-translate-client/resources/app.asar/app.html#/#)d by pepsin


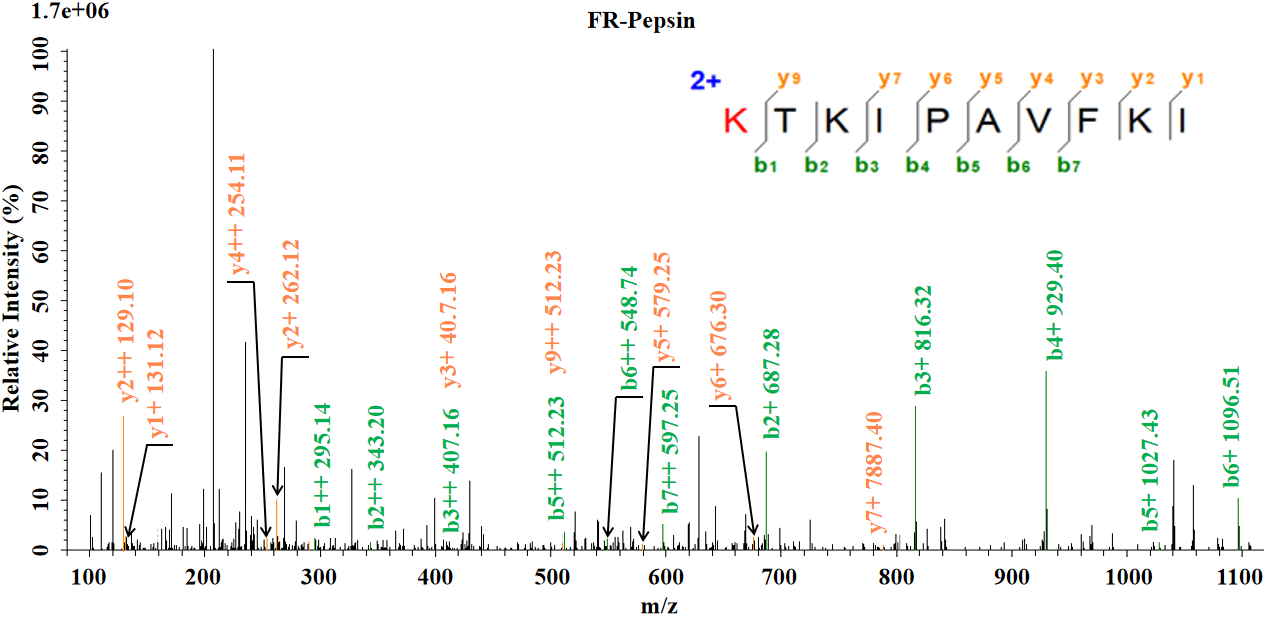


S Fig. 2e Peptide of KTKIPAVFKI in FR sample [hydrolyze](file:///C:/Program%20Files/baidu-translate-client/resources/app.asar/app.html#/#)d by pepsin


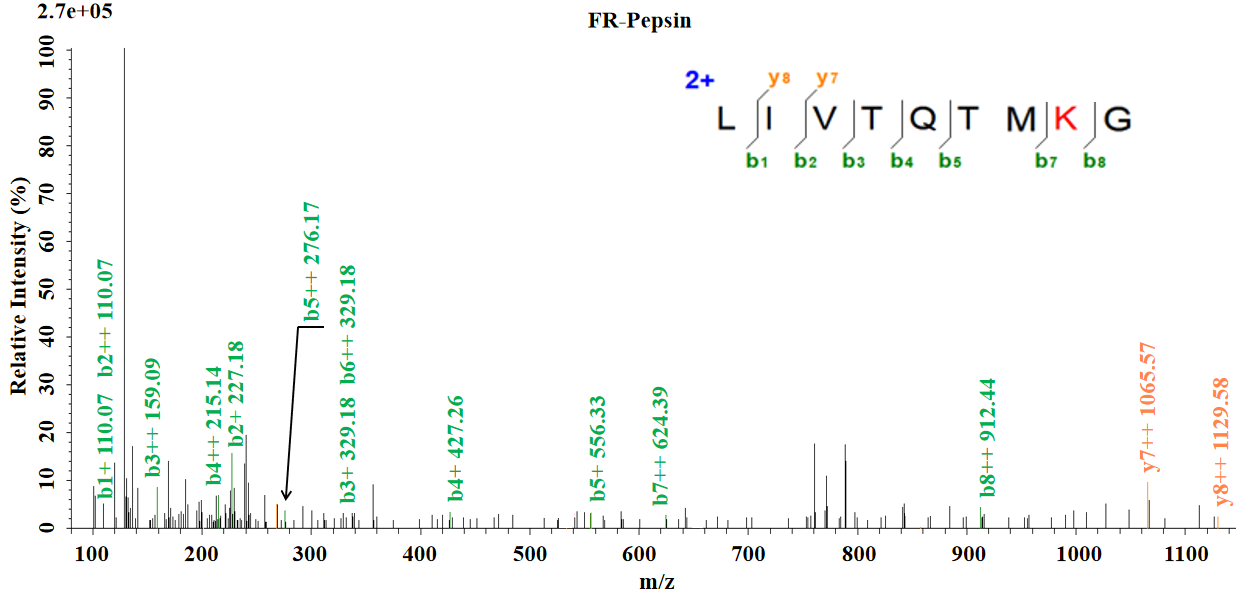


S Fig. 2f Peptide of LIVTQTMKG in FR sample [hydrolyze](file:///C:/Program%20Files/baidu-translate-client/resources/app.asar/app.html#/#)d by pepsin


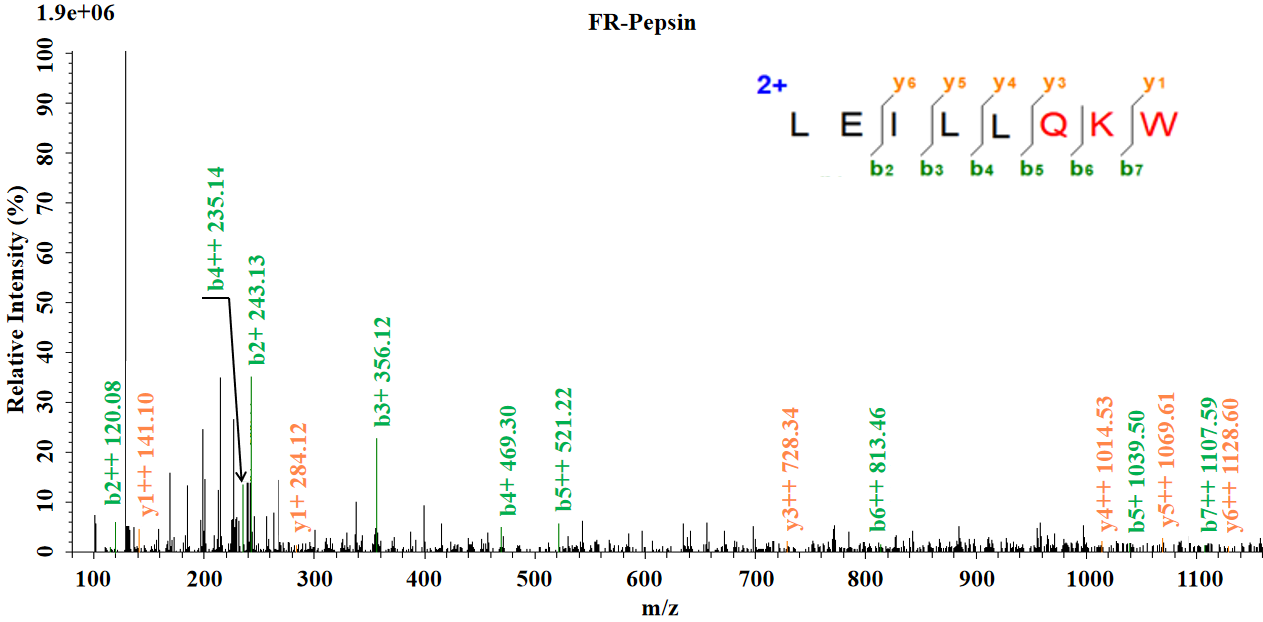


S Fig. 2g Peptide of LEILLQKW in FR sample [hydrolyze](file:///C:/Program%20Files/baidu-translate-client/resources/app.asar/app.html#/#)d by pepsin


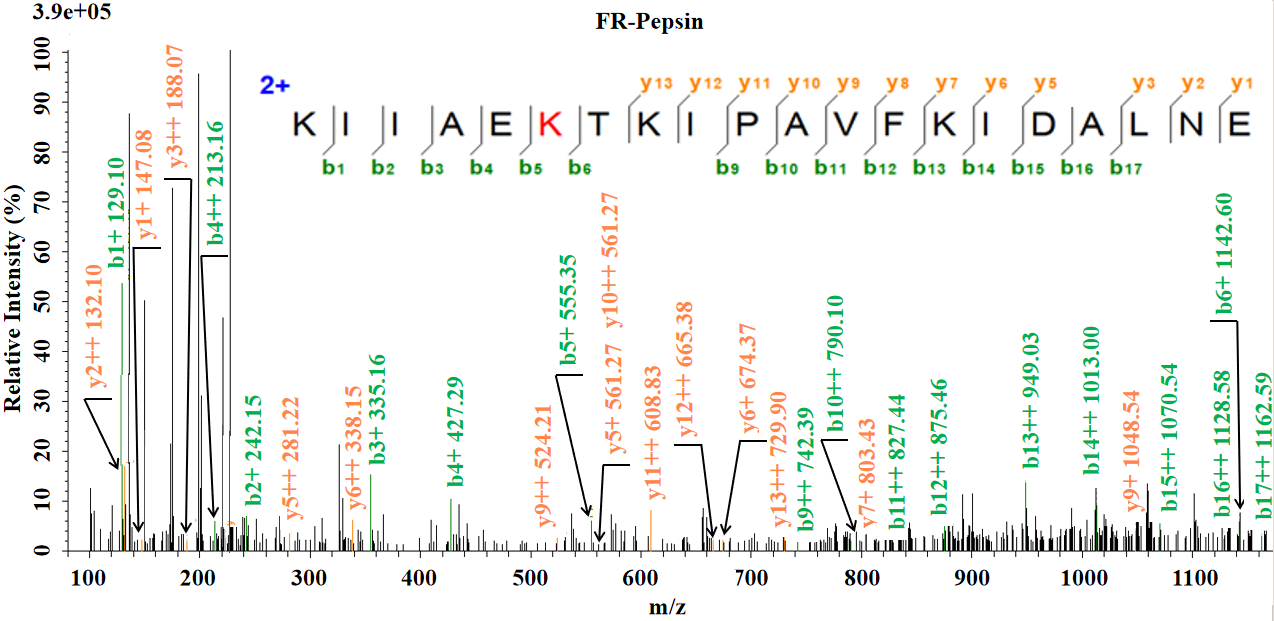


S Fig. 2h Peptide of KIIAEKTKIPAVFKIDALNE in FR sample [hydrolyze](file:///C:/Program%20Files/baidu-translate-client/resources/app.asar/app.html#/#)d by pepsin


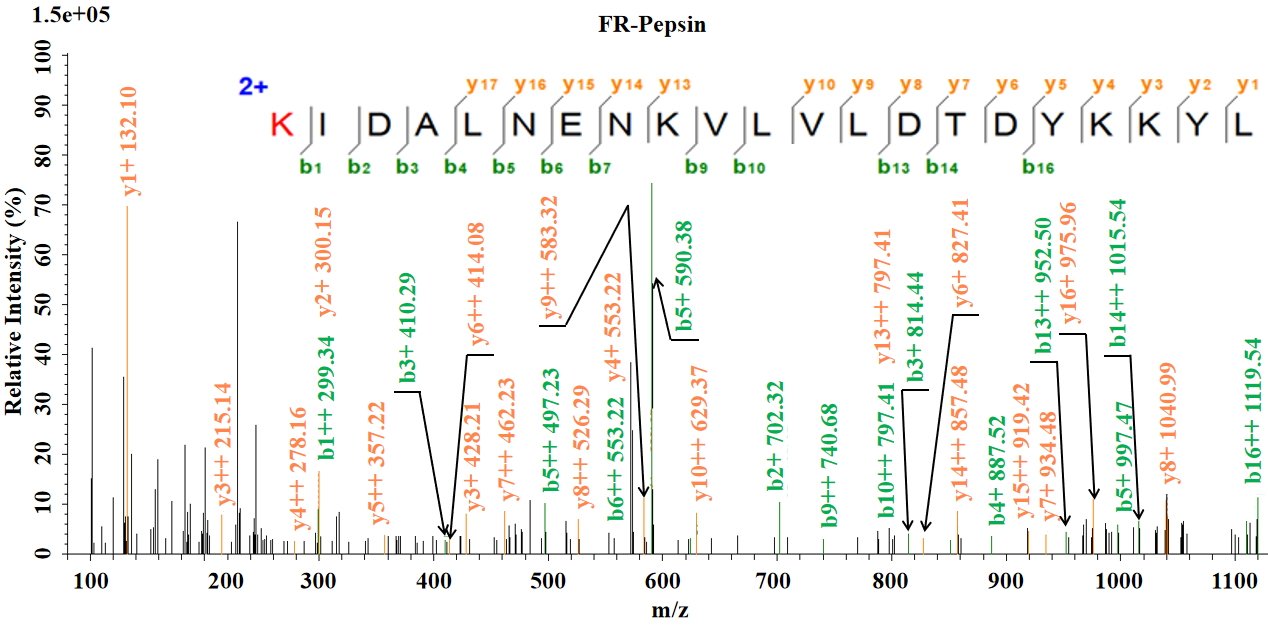


S Fig. 2i Peptide of KIDALNENKVLVLDTDYKKYL in FR sample [hydrolyze](file:///C:/Program%20Files/baidu-translate-client/resources/app.asar/app.html#/#)d by pepsin


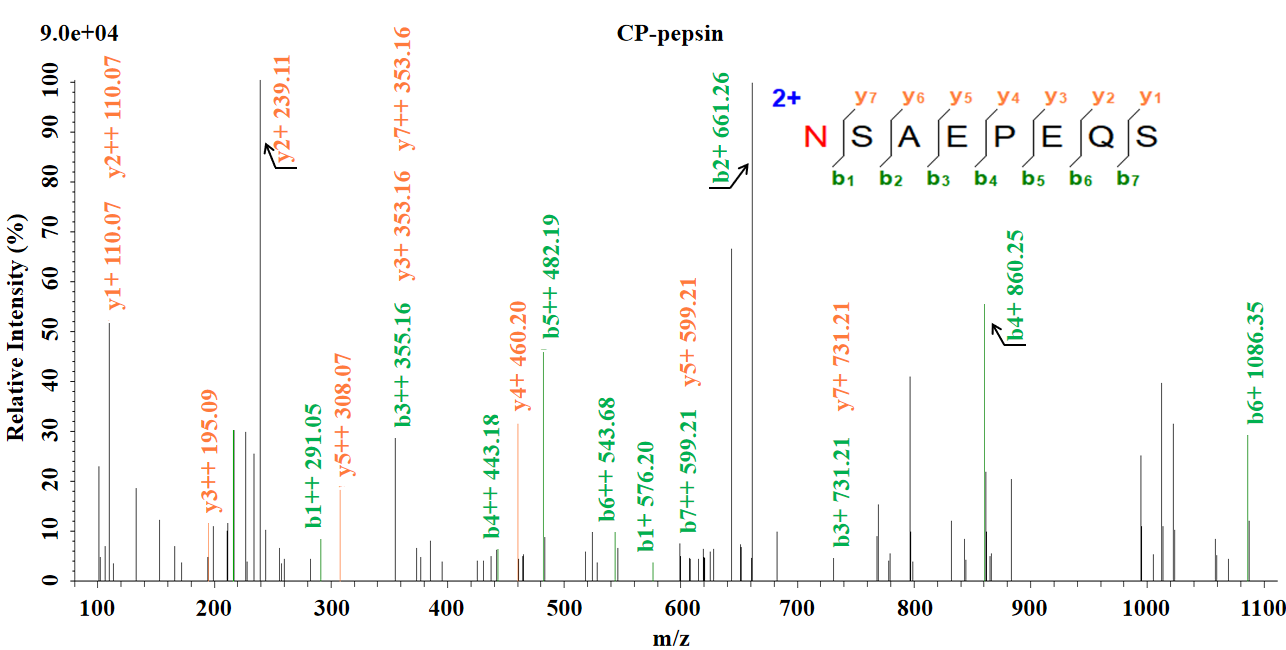


S Fig. 2j Peptide of NSAEPEQS in CP sample [hydrolyze](file:///C:/Program%20Files/baidu-translate-client/resources/app.asar/app.html#/#)d by pepsin


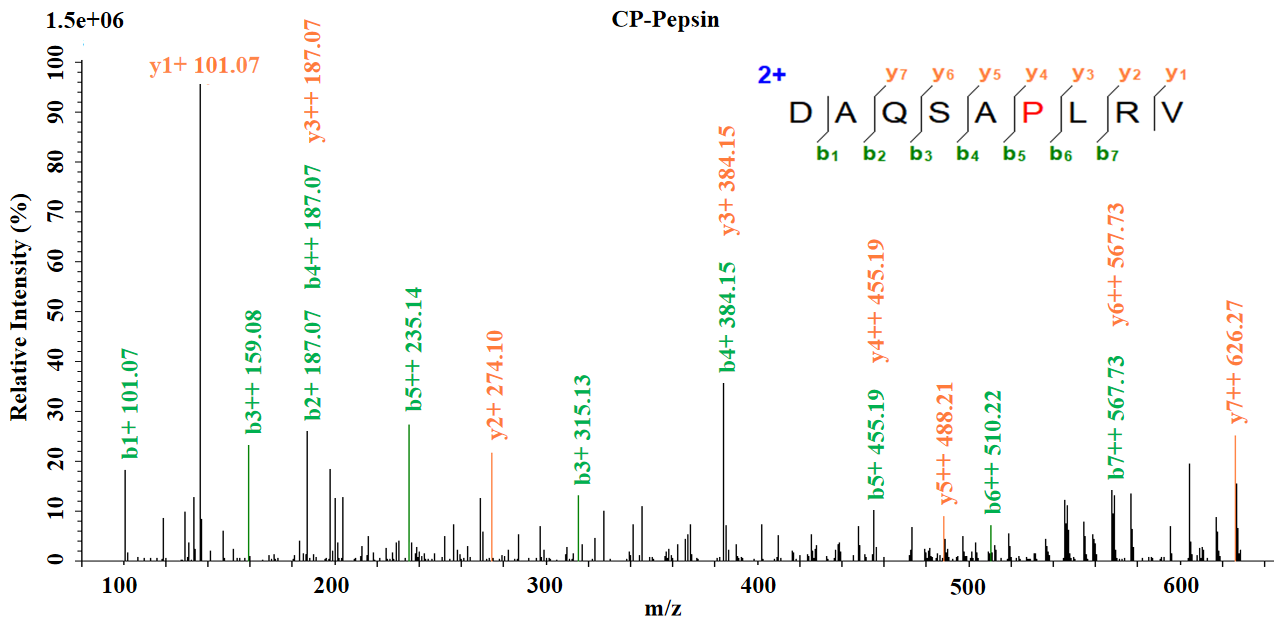


S Fig. 2k Peptide of DAQSAPLRV in CP sample [hydrolyze](file:///C:/Program%20Files/baidu-translate-client/resources/app.asar/app.html#/#)d by pepsin


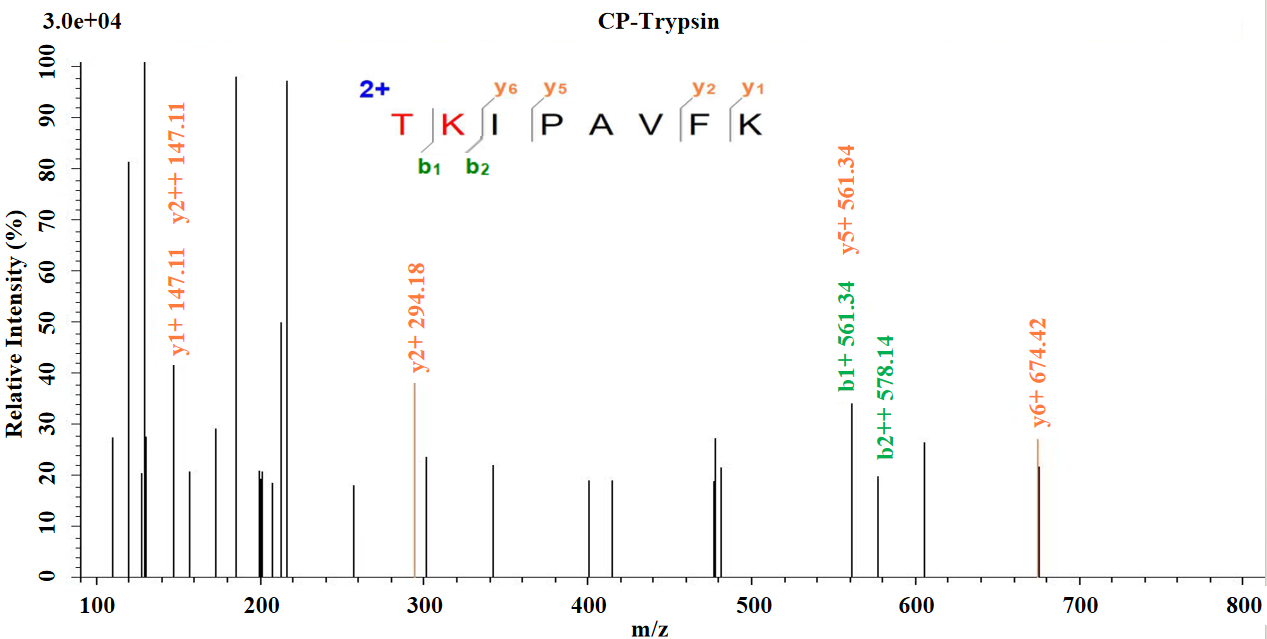


S Fig. 2l Peptide of TKIPAVFK in CP sample [hydrolyze](file:///C:/Program%20Files/baidu-translate-client/resources/app.asar/app.html#/#)d by trypsin


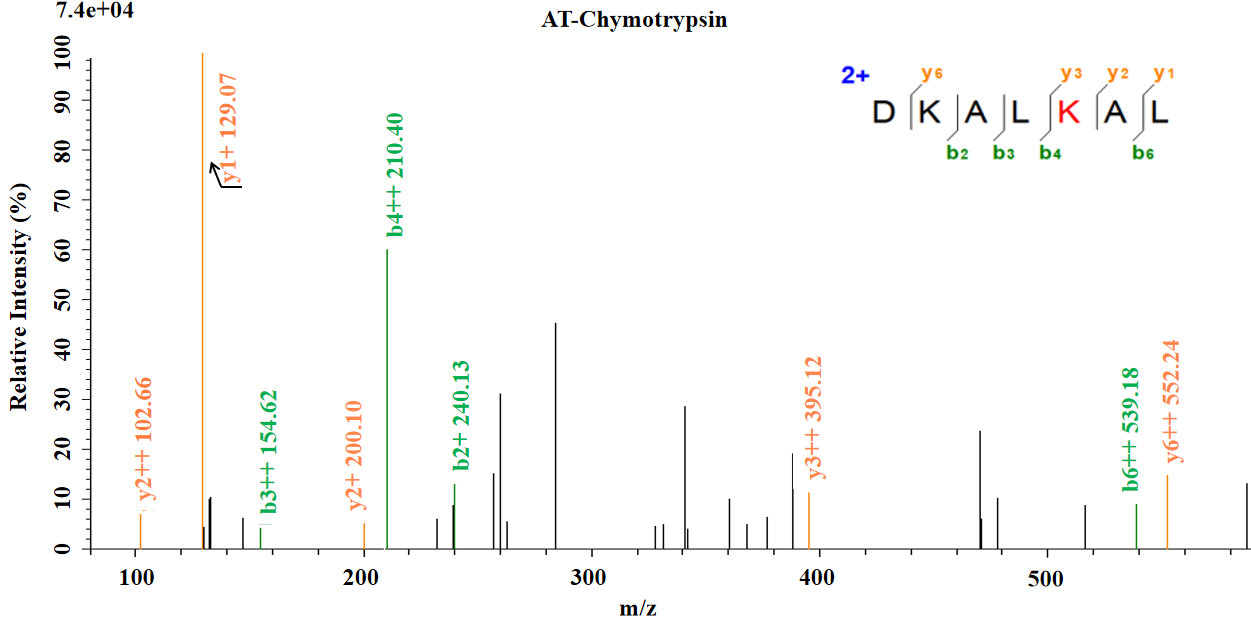


S Fig. 2m Peptide of DKALKAL in AT sample [hydrolyze](file:///C:/Program%20Files/baidu-translate-client/resources/app.asar/app.html#/#)d by chymotrypsin


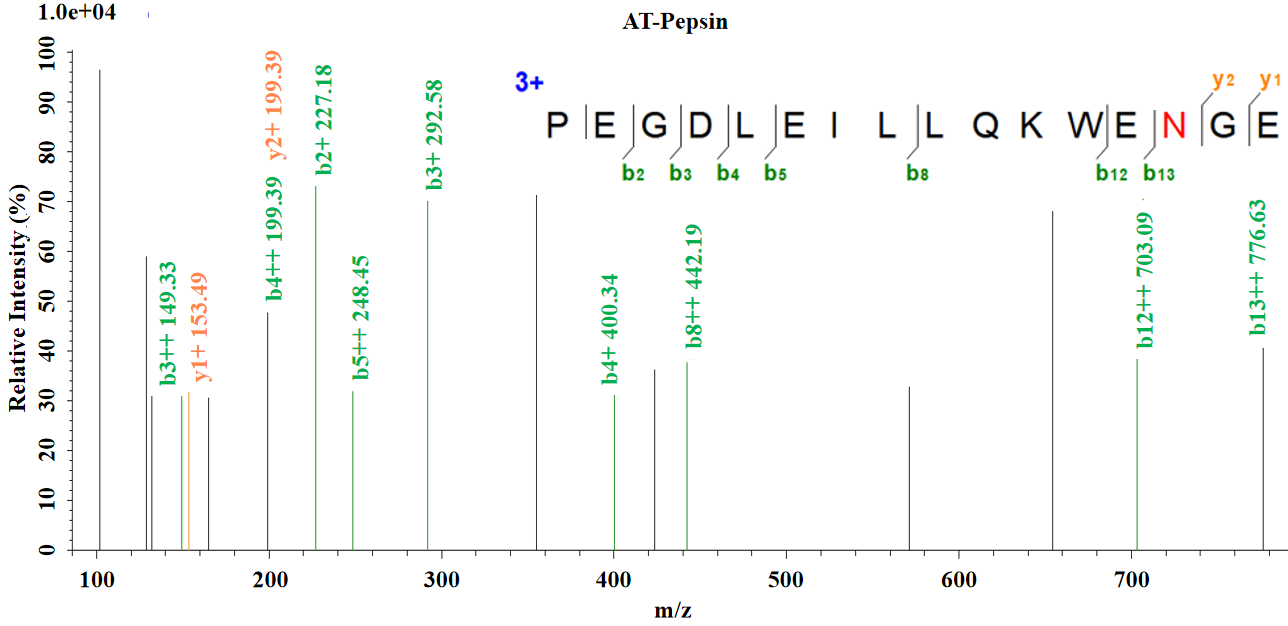


S Fig. 2n Peptide of PEGDLEILLQKWENGE in AT sample [hydrolyze](file:///C:/Program%20Files/baidu-translate-client/resources/app.asar/app.html#/#)d by pepsin
